# Group 3 innate lymphocytes make a distinct contribution to type 17 immunity in bladder defence

**DOI:** 10.1016/j.isci.2022.104660

**Published:** 2022-06-22

**Authors:** Alexandra M. Riding, Kevin W. Loudon, Andrew Guo, John R. Ferdinand, Laurence S.C. Lok, Nathan Richoz, Andrew Stewart, Tomas Castro-Dopico, Zewen Kelvin Tuong, Remi Fiancette, Georgina S. Bowyer, Aaron Fleming, Eleanor S. Gillman, Ondrej Suchanek, Krishnaa T. Mahbubani, Kourosh Saeb-Parsy, David Withers, Gordan Dougan, Simon Clare, Menna R. Clatworthy

**Affiliations:** 1 Molecular Immunity Unit, University of Cambridge Department of Medicine MRC Laboratory of Molecular Biology, Cambridge Biomedical Campus, Francis Crick Avenue, Cambridge CB2 0QH, UK; 2 Cambridge Institute for Therapeutic Immunology and Infectious Diseases (CITIID), Jeffrey Cheah Biomedical Centre, Cambridge Biomedical Campus, Puddicombe Way, Cambridge CB2 0AW, UK; 3 Cellular Generics, Wellcome Sanger Institute, Hinxton, UK; 4 Institute of Immunology and Immunotherapy, College of Medical and Dental Sciences, University of Birmingham, Birmingham, UK; 5 University of Cambridge Department of Surgery, Cambridge, UK; 6 NIHR Cambridge Biomedical Research Centre, Cambridge, UK; 7 Parasites and Microbes, Wellcome Sanger Institute, Hinxton, UK

**Keywords:** Immunology, Cell biology, Transcriptomics

## Abstract

Bladder infection affects a hundred million people annually, but our understanding of bladder immunity is incomplete. We found type 17 immune response genes among the most up-regulated networks in mouse bladder following uropathogenic *Escherichia coli* (UPEC) challenge. Intravital imaging revealed submucosal *Rorc*+ cells responsive to UPEC challenge, and we found increased *Il17* and *IL22* transcripts in wild-type and *Rag2*^−/−^ mice, implicating group 3 innate lymphoid cells (ILC3s) as a source of these cytokines. NCR-positive and negative ILC3 subsets were identified in murine and human bladders, with local proliferation increasing IL17-producing ILC3s post infection. ILC3s made a more limited contribution to bladder IL22, with prominent early induction of IL22 evident in Th17 cells. Single-cell RNA sequencing revealed bladder NCR-negative ILC3s as the source of IL17 and identified putative ILC3-myeloid cell interactions, including via lymphotoxin-β-LTBR. Altogether, our data provide important insights into the orchestration and execution of type 17 immunity in bladder defense.

## Highlights

• Type 17 immune gene network is the major transcriptional response post bladder infection
• Group 3 innate lymphoid cells (ILC3s) identified in mouse and human bladder
• ILC depletion in *Rag2*^*−/−*^ mice resulted in increased bladder bacterial load
• IL17 production by ILC3 shape bladder macrophage activation and polarization

## Graphical abstract

**Figure:**
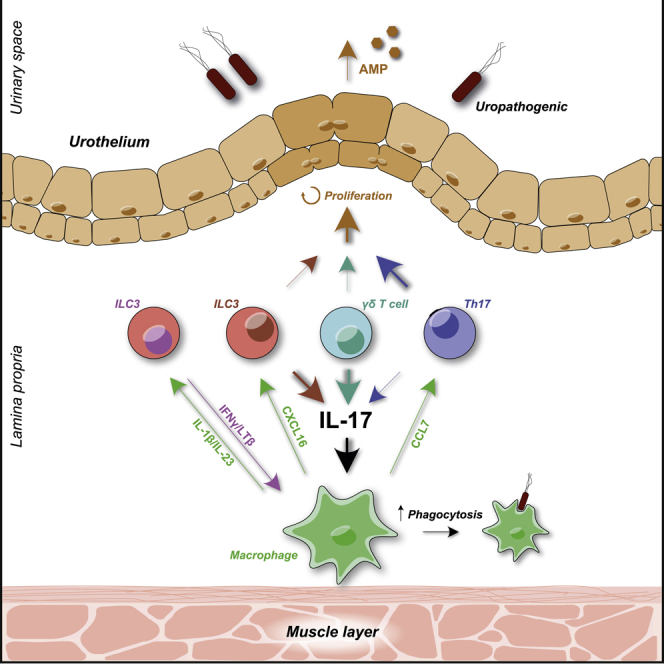

## Introduction

Bacterial infection of the lower renal tract (cystitis) is common, affecting half of women at some point in their lifetime, with significant associated socioeconomic cost (Chertow et al., 2005; Foxman, 2014). The majority of uncomplicated infections are caused by uropathogenic *Escherichia coli* (UPEC) that can also ascend to the kidneys causing pyelonephritis and renal scarring (Foxman, 2014; Svensson et al., 2011). Tissue-resident immune cells are vital for initiating and propagating local immune responses, but our understanding of the nature and function of these cells in the bladder, particularly in humans, is incomplete.

In recent years, innate lymphoid cells (ILCs) have been identified in a number of environment-facing organs, including the gastrointestinal tract, skin, and lung, as the innate counterparts of T cells, providing an early source of cytokines that have the potential to shape innate and adaptive immune responses (Klose and Artis, 2020; Panda and Colonna, 2019). Group 3 ILCs are dependent on the transcription factor RORγt for their development (Eberl et al., 2004; Sanos et al., 2009) and secrete canonical Th17 cytokines (IL22, IL17, and GM-CSF) in response to stimulation with myeloid cell-derived cytokines such as IL1β, IL23, and TL1A (Melo-Gonzalez and Hepworth, 2017). In the intestine ILC3-derived IL22 contributes to the maintenance of epithelial stem cells (Aparicio-Domingo et al., 2015; Lindemans et al., 2015) and increases epithelial production of anti-microbial peptides (AMPs) to protect against enteric pathogens (Ahlfors et al., 2014; Cella et al., 2009; Guo et al., 2014). Human primary urothelial cells have been shown to express both the membrane-associated IL22 receptor subunit, IL22RA1, and the secreted soluble form IL22RA2 and to respond to *ex vivo* IL22 stimulation by producing S100A9 and lipocalin-2 (LCN2) (Le et al., 2014), two important AMPs. However, the cellular sources of IL22 in the bladder have not been delineated, and the role of IL22 in bladder defense in the context of bacterial infection is unknown. In the intestine and lung, ILC3s also produce IL17 and GM-CSF in the context of infection or inflammation (Buonocore et al., 2010; Castro-Dopico et al., 2020; Mortha et al., 2014; Pearson et al., 2016; Van Maele et al., 2014). In the bladder, IL17 is protective in murine models of UPEC-associated cystitis, with γδT cells suggested as a major source of this cytokine (Sivick et al., 2010), in keeping with a previous study showing increased susceptibility of γδT cell-deficient mice to acute cystitis (Jones-Carson et al., 1999). Interestingly, higher IL17 levels in infected bladders of female compared with male mice was found to contribute to sex-based differences in susceptibility to chronic UPEC infection (Zychlinsky Scharff et al., 2019). Although the cellular source of IL17 was not delineated in this study, *Rag2*^−/−^γc^−/−^female mice were unable to clear bacteria from the bladder post challenge (Zychlinsky Scharff et al., 2019), in contrast to previous reports in *Rag2*^−/−^ (Mora-Bau et al., 2015), implicating ILC3s as potentially important players in bladder defense. The protective effects of IL17 more generally in infection beyond urinary tract infections (UTIs) are mediated by several mechanisms; IL23-induced IL17 can regulate granulopoiesis via G-CSF and increase peripheral neutrophilia (Parsonage et al., 2008; Stark et al., 2005), and IL17 also has important effects on monocyte recruitment and macrophage function (Silverpil et al., 2011; Veldhoen, 2017). IL17 can also act in concert with IL22 to stimulate AMP production by epithelial cells (Liang et al., 2006).

Here we sought to investigate type 17 immunity in bladder defense, because our unbiased transcriptomic analysis of UPEC-infected bladders identified this axis as one of the most up-regulated networks in acute bacterial infection. We validated an increase in bladder *Il17* and *IL22* transcripts in acute UPEC cystitis in both wild type (WT) and *Rag2*^−/−^ mice, implicating ILC3s in type 17 responses in the bladder. A network of *Rorc*+ cells were visualized in the submucosa using intravital, two-photon imaging, and were sessile in homeostasis, but showed increased motility post UPEC challenge. We confirmed that ILC3s were present in human and murine bladders, including both natural cytotoxicity receptor (NCR)+ and NCR negative (neg) subsets. *Rorc*-deficient mice had increased bacterial counts in the bladder, with reduced *Il17* and *Il22* transcripts, as well as a reduction in neutrophil recruitment and impaired monocyte differentiation into tissue macrophages. IL22R-deficient mice had no acute impairment of bacterial clearance but showed a reduction in bladder AMPs and in urothelial cell proliferation following UPEC challenge. ILC depletion in *Rag2*^*−/−*^ mice resulted in increased bladder bacterial load and a marked reduction in *Il17* transcripts and an increase in an M2 macrophage transcriptional signature in the bladder, emphasizing the importance of bladder ILC3s in shaping tissue macrophage polarization. Single-cell RNA sequencing of murine bladder post UPEC challenge confirmed the relative contributions of Th17, ILC3, and γδT cells to IL17, IL22, and GMC-CSF production in the bladder and also identified IFNγ and LTβ secretion as potential mediators of ILC3-macrophage interactions. Altogether, our data show distinct populations of tissue type 17 immune cells that are primed for rapid responses to bacterial challenge, orchestrating epithelial and myeloid cell function for bladder defense.

## Results

### Induction of type 17 immunity during bladder infection

**Figure 1:**
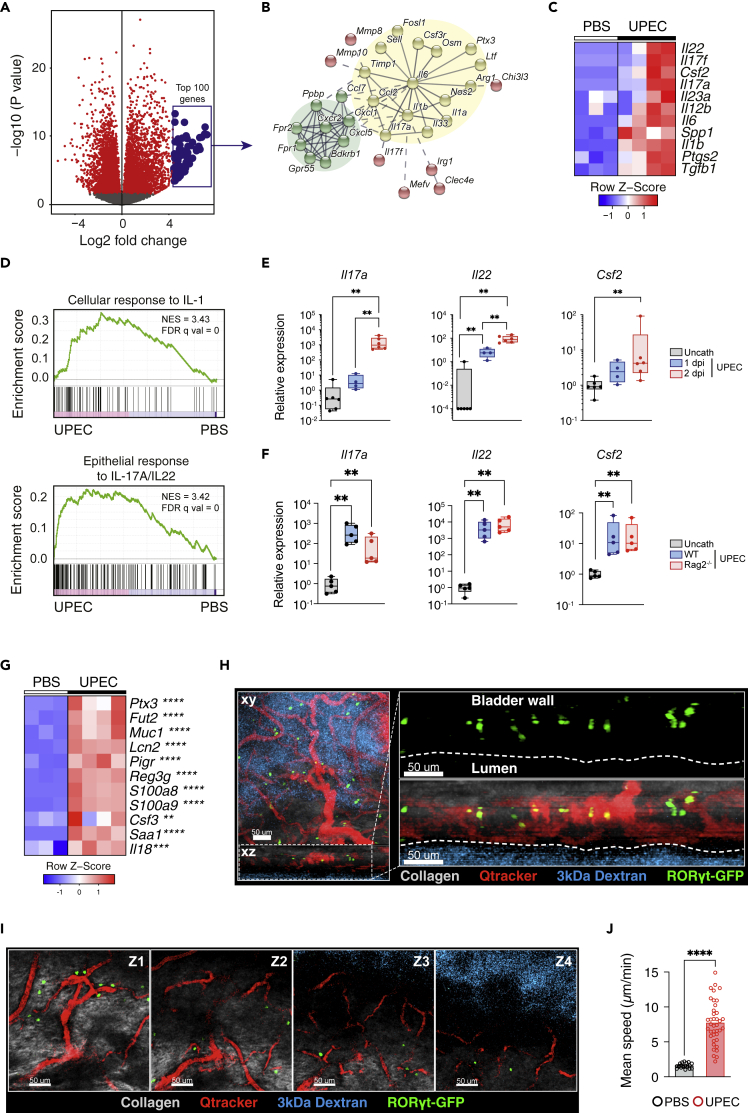
Induction of type 17 immunity during bladder infection (A) Volcano plot of up- and down-regulated genes in murine bladders 24 h after UTI. RNA-seq was performed on C57BL/6J bladders catheterized with either PBS (n = 3) or UTI89 (n = 4) and euthanized after 24 h. (B) STRING analysis (https://string-db.org) of top 100 up-regulated genes following UTI ranked by LFC. Th17 immunity and neutrophil recruitment nodes highlighted in yellow and green, respectively. (C) Heatmap of top 11 differentially expressed cytokines following UTI. (D) GSEA of IL1b (top panel) and IL17a/IL22 (bottom panel) response signatures in UTI from (A) (IL1b response: GO:0071347; IL17/22 response: M303, msigdb.org). (E) qPCR of Th17 cytokines in C57BL/6J bladders day 1 (blue) and day 2 post UTI (red) (n = 4-6 per group) relative to uncatheterized bladders (gray). (F) qPCR of Th17 cytokines in C57BL/6J (blue) and Rag^−/−^ (red) bladders day 1 post UTI (n = 4-6 per group) relative to uncatheterized bladders (gray). Data are representative of two independent experiments. Each point represents a single mouse bladder. (G) Heatmap of selected IL22-dependent AMPs from data in (A). (H and I) Intravital images of naïve Rorc^GFP^ murine bladder following intravenous Qtracker (blood vessels, red) and 3 kDa dextran-TMR (bladder lumen, cyan) labeling, with collagen in gray and RORγt^GFP^ cells green, showing location of RORγt^GFP^ cells (H) in submucosa in the x-z plane and (I) near blood vessels at different z-depths from submucosa (Z1) to bladder lumen (Z4). (J) Quantification of mean speed of RORγt^GFP^ cells in uninfected (black) and UPEC-infected (red) murine bladder, each point representing one cell track. Data are representative of two independent experiments. ∗p < 0.05, ∗∗p < 0.01, ∗∗∗p < 0.001, ∗∗∗∗p < 0.0001 by Mann-Whitney test (H), one-way ANOVA with Dunn’s multiple comparisons test (E-F), and two-way ANOVA with Šídák’s multiple comparisons test (C and G). All bladders used were from female mice unless otherwise stated.

To gain an unbiased insight into immunological pathways that potentially play a role in the local immune response to bladder infection, we challenged wild type (WT) C57BL/6 mice with UPEC and performed RNA sequencing (RNA-seq) on bladder tissues. We found that several thousands of genes were significantly up- and down-regulated following infection (Figure 1A). Analysis of the top 100 most up-regulated genes implicated two major interactions nodes; IL1β-associated Th17 immunity (Figures 1B and 1C) and, related to this, *Cxcl1*-driven neutrophil recruitment (Figure 1B). Gene set enrichment analysis confirmed an increase in genes associated with cellular responses to IL1 and in epithelial responses to IL17 and IL22 (Figure 1D). In an independent experiment and a previously published dataset (GEO:GSE68220), we confirmed a significant increase in *Il17*, *Il22,* and, to a lesser extent, *Csf2* transcripts, canonical Th17 cytokines, in the bladder in WT mice at early time points (24–48 h) following challenge with UPEC (Figures 1E and S1A), when adaptive immunity would not be expected to make a major contribution to the immune response. *Il17* and *Il22* were also increased in the bladders of *Rag2*^−/−^ mice that are deficient in γδ T cells and Th17 cells (Figure 1F), suggesting that ILC3s can act as a source of these cytokines during bacterial infection in the bladder.

In the gut, ILC3-derived cytokines can stimulate epithelial cells to produce anti-microbial peptides (AMPs) (Ahlfors et al., 2014; Guo et al., 2014). In the bladder, several AMPs have been shown to be induced during infection, including defensins (Hiratsuka et al., 2000), Tamm Horsfall protein (Saemann et al., 2005), lipocalin2 (Steigedal et al., 2014), and cathelicidin (Chromek et al., 2006). We observed an increase in a number of IL22-dependent AMPs, including Reg3γ and Lcn2 post UPEC challenge (Figure 1G). Interestingly, *Il18* was also up-regulated in bladders in this context (Figure 1G), a cytokine known to promote an IFNγ-producing phenotype in gastrointestinal ILC3 (Klose et al., 2013; Vonarbourg et al., 2010).

IL17 promotes immune cell infiltration into tissues both via the induction of monocyte and neutrophil recruiting chemokines (Veldhoen, 2017) and also the secretion of matrix metalloproteinases (MMPs), including MMP1, 2, 3, 8, 9, and 13 (Koenders et al., 2005), which make the extracellular matrix more accessible to incoming cells. We observed an increase in *Cxcl1*, *Cxcl5,* and *Ccl1* in bladder post UTI (Figure 1B), as well as in *Mmp3* and *Mmp9* (Figure S1B), consistent with increased IL17 activity.

Intravital imaging using a GFP reporter mouse for the canonical type 17 immune cell transcriptional factor Rorc demonstrated the presence of many *Rorc*+ cells in the bladder, particularly within the submucosa (Figure 1H), that were frequently located adjacent to blood vessels (Figures 1H, 1I, and S1C and Video S1). These cells were largely sessile in homeostasis but showed increased movement following UPEC challenge (Figures 1J and S1D and Video S2). Together, these data show that the bladder is prearmed and populated with a network of cells capable of mounting a rapid type 17 response following bacterial challenge.

Video S1 [related to figure 1] - RORγt+ cells are located in bladder submucosa adjacent to blood vessels

Video S2 [related to figure 1] - RORγt+ cells show increased movement following UPEC infection

### NCR^+^ and NCR^−^ ILC3s present in murine and human bladder and increase during infection

**Figure 2:**
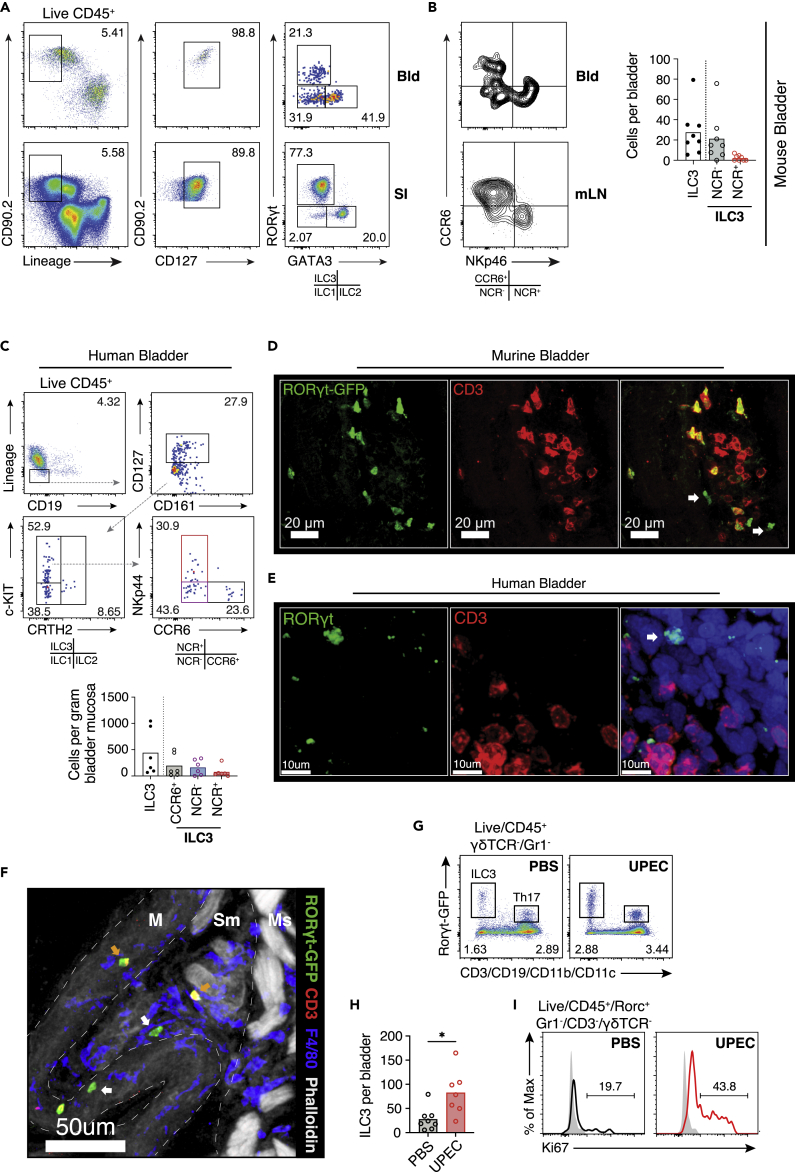
ILC3s present in murine and human bladder and increase during infection (A) Gating strategy for ILCs in naive C57BL/6J bladders (Bld) and small intestine (SI). Flow cytometry plots shown represent three pooled bladders. (B) Flow cytometry profiling of Ccr6 and NKp46 positivity on ILC3s in naive Rorcγt^GFP^ bladders and mesenteric lymph node (mLN) (left) and quantification of absolute cells counts for the indicated subsets (right). N=8 mice per group. Data are representative of two independent experiments. (C) Gating strategy for ILCs in n = 6 human bladder samples (top) and quantification of absolute cell counts per gram of tissue for the indicated subsets (bottom). Interrupted arrow (gray) denotes direction of gating. Colored flow gates correspond to subsets in graph beneath. Each dot represents an individual donor bladder (S2B). For absolute cell counts, medians are indicated. (D) Representative confocal image (n = 3) of naïve bladder from Rorcγt^GFP^ (male) mouse at 40× (green, Rorcγt^GFP^; red, CD3). (E) Confocal image of human female bladder from deceased donor at 40× (green, RORC; red, CD3; blue, DAPI). White arrows in (D) and (E) denote ILC3s. Donor details described in S2C. (F) A 40× confocal image of Rorcγt^GFP^ mouse (male) bladder showing localization of ILC3s (white arrow) to the submucosa (green, Rorcγt^GFP^; red, CD3; blue, DAPI; white, phalloidin). Structurally relevant areas are divided by an interrupted white line—mucosa (M), submucosa (SM), and muscularis (Ms). (G) Representative flow cytometry plots of ILC3s and Th17 cells in bladders of Rorcγt^GFP^ reporter mice following catheterization with PBS or UTI89 (combination of three bladders per condition). (H) Quantification of ILC3 cell counts per bladder following PBS or UTI89 24 hours post infection. N = 7-8 mice per group. Data are representative of two independent experiments. (I) Percentage Ki67 expression by bladder ILC3s following PBS or UTI89 24 h post infection. N = 3 bladders were pooled per condition with three biological replicates. PBS, black; UPEC, red; and Isotype control, filled gray. Number denotes percentage positive Ki67. ∗p < 0.05, ∗∗p < 0.01, ∗∗∗p < 0.001 by Mann-Whitney test. All bladders used were from female mice unless otherwise stated.

Th17 cell and γδ T cells have previously been described in the bladder (Sivick et al., 2010), but given the increase in *Il17* and *Il22* transcripts at early time points post infection, even in T cell-deficient mice, we sought to characterize bladder ILC3s, as previous reports had described only CD4^+^ ILC3 (Zychlinsky Scharff et al., 2019). Flow cytometric analysis of tissue homogenates identified lineage negative (Lin^neg^), CD127+RORγt+ ILC3 in murine bladders, as well as GATA3+ ILC2 (Figures 2A, S2A and S2B). ILC3s can be further subdivided into lymphoid tissue-inducer (LTi)-like cells and natural cytotoxicity receptor (NCR)-positive subsets based on surface marker expression (Melo-Gonzalez and Hepworth, 2017). In human and murine bladder we identified both NCR^+^ and NCR^neg^ subsets (Figures 2B, 2C, and S2B–S2D). Confocal imaging confirmed a network of RORγt-positive cells in mouse and human bladder, which included CD3^neg^ ILC3s (Figures 2D, 2E and S2D–S2F). *The bladder comprises a number of layers*; *from the outer muscularis through to the mucosa*, *submucosal muscularis*, *lamina propria, and inner urothelium*. *We found that* ILC3s were localized to the submucosal lamina propria (Figure 2F). Following intravesical UPEC challenge, we observed an increase in ILC3 numbers (Figures 2G and 2H), suggesting either tissue recruitment or local proliferation. Consistent with the latter, there was a significant increase in the proportion of Ki67+ ILC3s evident in the bladder post infection (Figure 2I). There was also an increase in Th17 cell and γδ T cells post UTI (Figure S2G).

### *Rorc*-deficient mice have increased susceptibility to bladder infection

**Figure 3:**
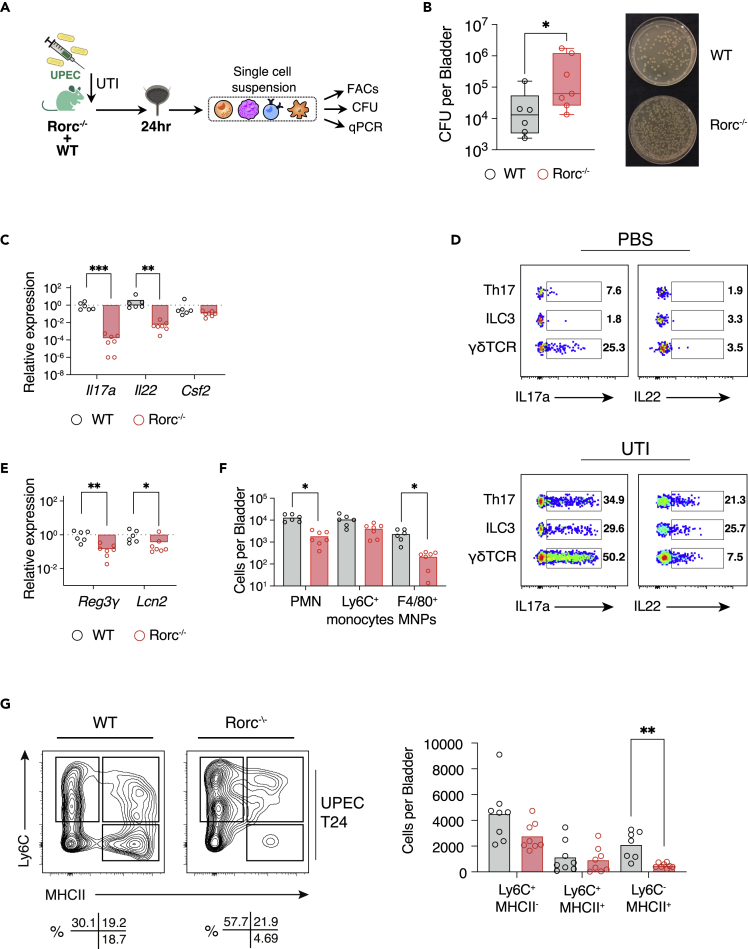
*Rorc*-deficient mice have increased susceptibility to bladder infection (A) Schematic of experimental design. (B) Colony-forming units per bladder in C57BL/6J (gray) and Rorc^−/−^ (red) bladders 24 h post UTI (left panel) (Table S1) and corresponding image of bacterial growth on agar plates; 1:30 dilution (right panel). N = 6-7 mice per group. (C) qPCR of Th17 cytokines in C57BL/6J (gray) and Rorc^−/−^ (red) bladders 24 h post infection. Results in (C) relative to C57BL/6J. Data are representative of two independent experiments. (D) Flow cytometry analysis of percentage expression of intracellular IL17a and IL22 in C57BL/6J bladders catheterized with PBS or UTI89. (E) qPCR of selected AMPs in C57BL/6J (gray) and Rorc^−/−^ (red) bladders 24 h post infection (n = 6-7 per group). Results relative to wild type. Data are representative of two independent experiments. (F) Quantification of cells counts for indicated subsets. (G) Bladder “monocyte waterfall” subset quantification by flow cytometry 24 h post infection with UTI89 in C57BL/6J and Rorc^−/−^ bladders (n = 8 per group). Flow plots of CD45+Ly6G-CD11b+CX3CR1+ waterfall subsets (left) and quantification of absolute cell counts for the indicated subsets (right) are shown. ∗p < 0.05, ∗∗p < 0.01, ∗∗∗p < 0.001 by Mann-Whitney test (B, E) and one-way ANOVA with Dunn’s multiple comparisons test (C, F-G). All bladders used were from female mice unless otherwise stated.

We next sought to address the functional importance of type 17 immunity in bladder defense using *Rorc*-deficient mice that lack ILC3, as well as γδT cells and Th17 cells, and therefore have a significantly diminished capacity to produce Th17 cytokines (Figures 3A and S3A). At 48 h following intravesical UPEC challenge, we observed a significant increase in bladder colony forming units (CFUs) in *Rorc*-deficient mice relative to WT mice (Figures 3B and S3A and Table S1). *Il17* and *Il22* transcripts were reduced in *Rorc*^−/−^ bladders following infection compared with controls, with similar levels of *Csf2* (Figure 3C). Intracellular cytokine staining in WT bladders following *in vitro* stimulation identified IL17 production in γδ T cells in both homeostasis and infection (Figures 3D and S3B), implying that they provide a basal level of defense. In contrast, there was little cytokine production evident in Th17 cells in homeostasis, but a marked induction of IL22 and IL17 (Figures 3D and S3B), even at this early time point post infection before a primary adaptive response would have time to occur, suggestive of a recall response. There was also little basal cytokine production in ILC3s but a 20-fold increase in the proportion of IL17-producing ILC3s post UPEC challenge, with a more modest increase in IL22 production (Figures 3D and S3B). Similarly, these cytokines were increased in whole bladder lysates post UPEC challenge by ELISA (S3C). This pattern of cytokine production is in sharp contrast to ILC3s in the gastrointestinal tract that make little contribution to tissue IL17 during inflammation, but rather act as an early source of IL22 (Lee et al., 2015).

In the gut IL22 stimulates AMP production by intestinal epithelial cells to promote barrier integrity (Ahlfors et al., 2014; Cash et al., 2006; Guo et al., 2014; Sano et al., 2015). Consistent with a similar paradigm in bladder, we observed a significant reduction in *Lcn2* and Reg3γ (Figure 3E). In keeping with the known effects of IL17, there was a reduction in immune cell infiltrates into the bladder in *Rorc*−/− mice, particularly neutrophils, and also F4/80+ macrophages (Figure 3F). Infiltrating monocytes entering tissues progressively lose Ly6C expression and increase MHC-II expression as they become tissue macrophages, the so-called waterfall phenomenon, first described in the intestine (Tamoutounour et al., 2012) (Mora-Bau et al., 2015). In *Rorc*−/− mice, Ly6C+ MHCII^neg^ cell numbers were similar to WT counterparts but we observed a reduction in MHCII+ cells, consistent with an impaired maturation of infiltrating monocytes (Figure 3G).

Together, these data show that type 17 immune cells are important for coordinated defense in the bladder early in the time course of infection, enabling myeloid cell infiltration and maturation, as well as epithelial production of AMPs.

### IL22 promotes epithelial proliferation and AMP production in the bladder post infection

**Figure 4:**
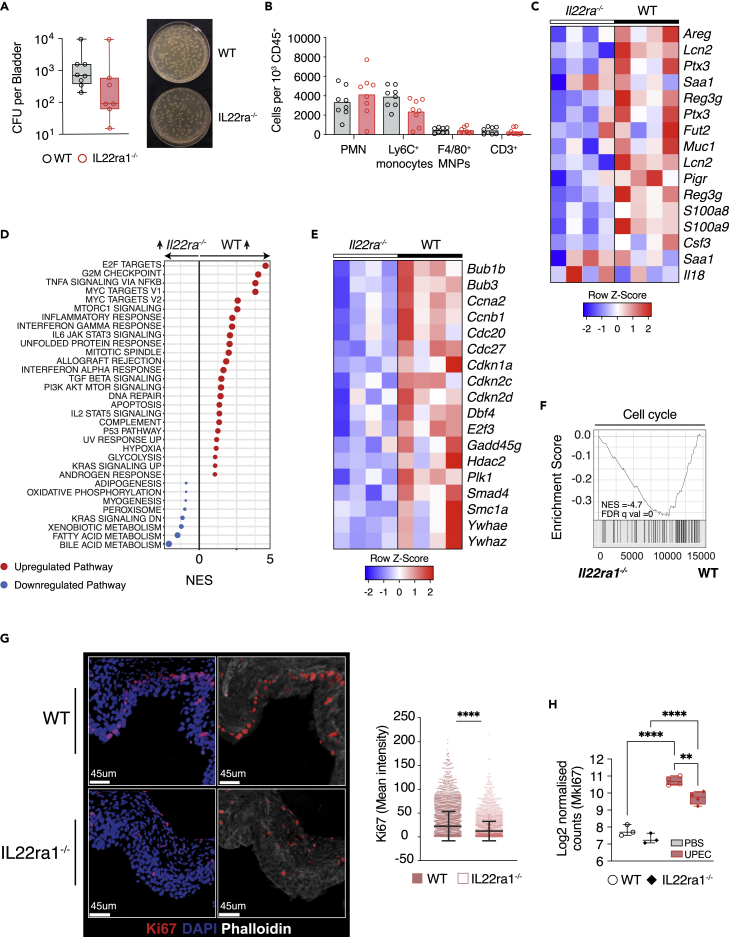
IL22 promotes epithelial AMP production in the bladder post infection (A and B) (A) Colony-forming units per bladder and (B) quantification of cells counts (per 1 × 10^3^ CD45^+^ cells) for indicated subsets 24 h after challenge in C57BL/6N (gray) or IL22ra^−/−^ (red) mice infected with UTI89 (left panel) (Table S1) and corresponding image of bacterial growth on agar plates; 1:30 dilution (right panel). N = 7-8 mice per group. (C) Heatmap of AMPs from RNA sequencing of bladders infected with UTI89 in C57BL/6N (n = 4) or IL22ra^−/−^ (n = 4) mice 24 h after challenge. Data represent four biological replicates per group (IL22ra^−/−^ and C57BL/6N). (D) Gene set enrichment analysis (www.gsea-msigdb.org/gsea) of the differential expression from (C) against hallmarks pathways. Only significant pathways (false discovery rate [FDR] q value < 0.05) are plotted. Red dots indicate positive enrichment and blue negative, the size of the dot is inversely correlated with the FDR q value and the position indicates the normalized enrichment score (NES). (E) Heatmap of cell cycle gene transcripts from data in (C). (F) Enrichment plot from GSEA for “cell cycle” pathways from the Kegg database. (G) Representative confocal image of infected bladders from C57BL/6N or IL22ra^−/−^ mice 24 h post infection (left) (red, Ki67; blue, DAPI and white, phalloidin) and quantification of Ki67 mean intensity fluorescence; each square (red filled, C57BL/6N; red open, IL22ra^−/−^) represents a cell surface (right). Mean ± SEM is shown. (H) Mki67 Log normalized counts from data in (C). ∗p < 0.05, ∗∗p < 0.01, ∗∗∗p < 0.001, ∗∗∗∗p < 0.0001 by Mann-Whitney test (A, G), one-way ANOVA with Šídák’s multiple comparisons test (B, H), and two-way ANOVA with Dunn’s multiple comparisons test (C, E). All bladders used were from female mice unless otherwise stated.

To further explore the effect of IL22 on the bladder epithelium, we challenged IL22RA1-deficient mice with UPEC. There were no differences in bladder CFUs at 24 or 48 h between WT and *IL22ra1*^−/−^ mice (Figures 4A, S4A–S4B and Table S1), and similar immune cell infiltration was observed (Figure 4B). However, there was a significant attenuation of AMP transcripts in *IL22ra1*^−/−^ bladders post infection (Figure 4C). Gene set enrichment analysis (GSEA) of bladder bulk RNA-seq data indicated a significant enrichment in *E2F target* genes and *G2M checkpoint* pathway genes in WT compared with *IL22ra1*^−/−^ bladders (Figure 4D). E2F transcription factors are important regulators of genes involved in G1 to S-phase progression, many of which showed decreased expression in *IL22ra1*^−/−^ bladders (Figures 4E and 4F). Consistent with an important role for IL22 in epithelial barrier repair in the bladder, we observed fewer Ki67-positive cells within the bladder epithelium in *IL22ra1*^−/−^ mice at 24 h following infection (Figure 4G) as well as a reduction in *Mki67* transcripts (Figure 4H).

### ILC depletion in *Rag2*^−/−^ mice leads to reduced *Il17* and increased infection severity

**Figure 5:**
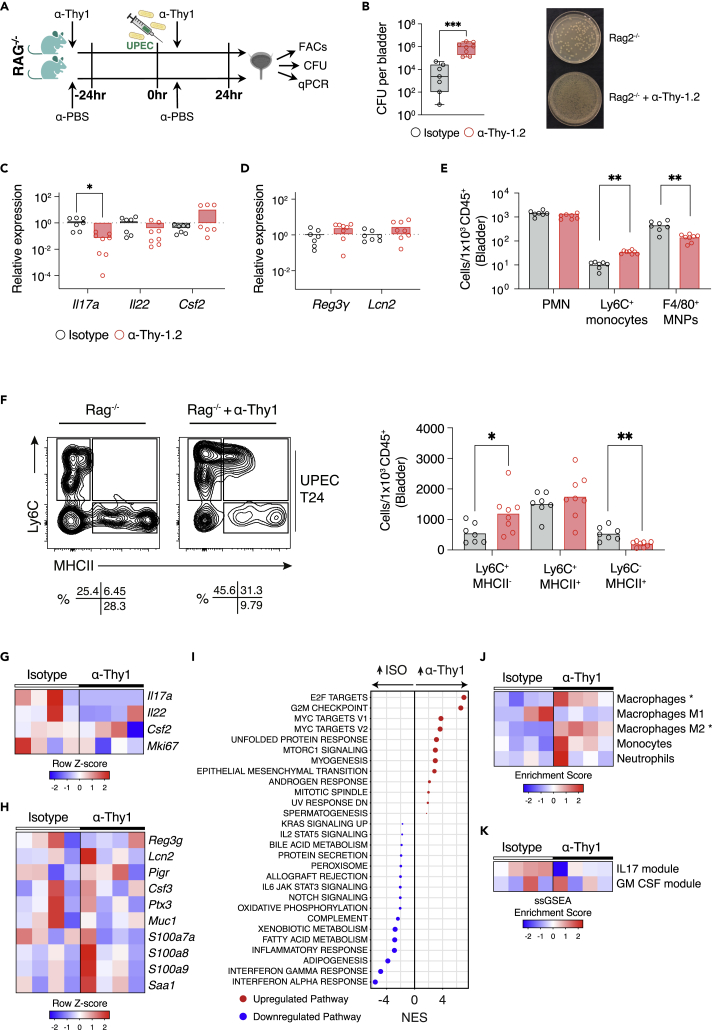
ILC depletion in *Rag2*^−/−^ mice increases the severity of cystitis (A) Schematic of experimental design. (B) Colony-forming units per bladder 24 h after infection with UTI89 in *Rag2*^*−/−*^ + isotype (gray) and *Rag2*^*−/−*^ + anti-Thy1 (red) mice (left panel) (Table S1) and corresponding image of bacterial growth on agar plates; 1:400 dilution (right panel). N = 7-8 mice per group. (C and D) Corresponding qPCR of Th17 cytokines (C) and selected AMPs (D) in *Rag2*^*−/−*^ + isotype (gray) and *Rag2*^*−/−*^ + anti-Thy1 (red) bladders 24 h post infection (n = 6 per group). Results relative to *Rag2*^*−/−*^ bladders. Data are representative of three independent experiments. (E) Quantification of absolute cell counts in *Rag2*^*−/−*^ + isotype (gray) and *Rag2*^*−/−*^ + anti-Thy1 (red) bladders 24 h post infection (n = 7-8 per group) for the indicated subsets. (F) Bladder “monocyte waterfall” subset quantification by flow cytometry 24 h post infection with UTI89 in *Rag2*^*−/−*^ + isotype and *Rag2*^*−/−*^ + anti-Thy1 bladders (n = 7-8 per group). Flow plots of CD45+Ly6G-CD11b+CX3CR1+ waterfall subsets (left) and quantification of absolute cell counts for the indicated subsets (right) are shown. (G) Heatmap of Th17 cytokines from RNA sequencing of bladders infected with UTI89 in *Rag2*^*−/−*^ + isotype (n = 4) and *Rag2*^*−/−*^ + anti-Thy1 (n = 4) female mice 24 h after challenge. Data represent four biological replicates per group (isotype and anti-Thy1). (H) Heatmap of selected IL22-dependent AMPs from (G). (I) Gene set enrichment analysis of the differential expression from (G) against hallmarks pathways. Only significant pathways (FDR q value < 0.05) are plotted. Red dots indicate positive enrichment and blue negative, the size of the dot is inversely correlated with the FDR q value and the position indicates the normalized enrichment score (NES). (J) Heatmap of cellular deconvolution of data in (G) using xCell (https://xcell.ucsf.edu). Scaled enrichment score is plotted (blue-red) with greatest enrichment in red. (K) Heatmap of scaled enrichment scores from single-sample gene set enrichment analysis (ssGSEA, https://www.genepattern.org/modules/docs/ssGSEAProjection/4) of data in (G) for IL17a and GM-CSF signatures (up-regulated genes, p < 0.05, LFC>1.5). IL17a and GM-CSF signatures are derived from GEO: GSE20087 (Zhang et al, 2010) and GEO: GSE95404 (Zhang et al, 2010), respectively. ∗p < 0.05, ∗∗p < 0.01, ∗∗∗p < 0.001 by Mann-Whitney test (B, D-E), one-way ANOVA with Dunn’s multiple comparisons test (C, F) and two-way ANOVA with Šídák’s multiple comparisons test (H, J, and K). All bladders used were from female mice unless otherwise stated.

To investigate whether ILC3s play a significant role in bacterial defense in the bladder, and to further probe their contribution to epithelial-maintaining IL22 production versus IL17 production in the absence of T cells, we challenged *Rag2*^−/−^ mice with UPEC following ILC depletion with an anti-Thy1 antibody (Figures 5A and S5A–S5C). We observed a significant increase in CFUs in ILC-depleted mice post infection, consistent with an important role in bladder defense (Figures 5B, S5D and Table S1). Notably, ILC-deficient mice had a significant reduction in bladder *Il17* transcripts, as observed in the *Rorc*^−/−^ mice, but in contrast to *Rorc*^−/−^ mice, we observed little difference in bladder *Il22* in the absence of ILC3 (Figure 5C), suggesting that the major contribution of ILC3s is in IL17 production rather than IL22. In keeping with this conclusion, there was a similar abundance of bladder AMP transcripts in anti-Thy1-treated and control mice (Figure 5D). Neutrophil infiltration was also similar in WT and ILC-depleted mice, but there was a significant reduction in F4/80^hi^ macrophages in the latter (Figure 5E). Ly6C^hi^ monocytes were increased in ILC-depleted mice, with reduced MHCII^hi^ cells, again suggestive of impaired progression of monocytes down the “waterfall” (Figure 5F), as we had observed in *Rorc*^−/−^ mice.

To further understand the role of ILCs in bladder defense, we performed RNA-seq on bladder samples taken from *Rag2*^−/−^ mice treated with isotype control or anti-Thy-1 antibody at 24 h following infection. *Il17* transcripts were significantly reduced in ILC-depleted bladders, and there was also a more variable reduction in *Il22* (Figure 5G), and correspondingly little difference in AMP transcripts (Figure 5H). GSEA showed a reduction in a number of inflammatory pathways in the absence of ILCs, including *Interferon gamma response* and *Interferon alpha response* pathway genes, while cell proliferation-associated pathways were increased (Figure 5I). Cellular deconvolution indicated an increase in M2 macrophage-associated genes in ILC-depleted bladders, in keeping with a reduction in IFN-γ stimulation (Figure 5J) and conversely, enrichment for an IL17-stimulated macrophage gene signature in isotype-treated compared with anti-Thy1-treated bladders (Figure 5K).

Altogether, these data indicate that, in contrast to intestinal ILC3s, IL22-mediated epithelial cross talk is a less important part of their function in the context of bacterial challenge in the bladder; rather, IL17-mediated myeloid cell stimulation plays a more prominent role in this context.

### Bladder macrophages produce ILC3-stimulating cytokines during infection

**Figure 6:**
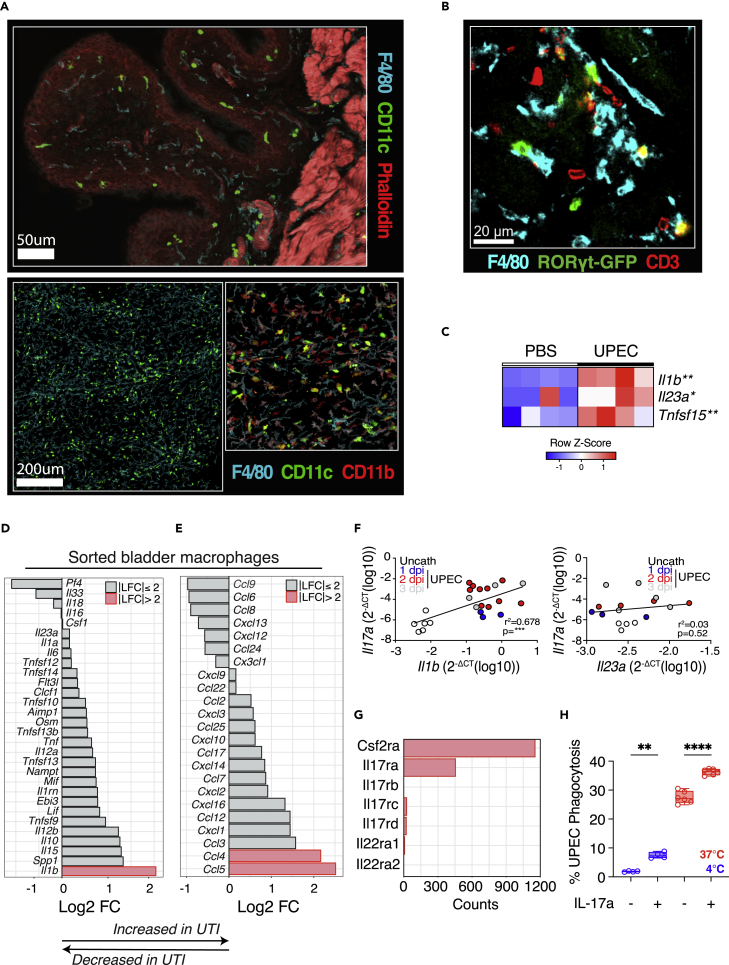
Bladder macrophages produce ILC3-stimulating cytokines during infection (A) Representative confocal image of naive murine bladder at 40× (upper panel) and whole mount (lower panel) (cyan, F4/80; green, CD11c; red, phalloidin). (B) Confocal image of naive bladder from Rorcγt^GFP^ mouse at 40× (blue, F4/80; green, Rorcγt^GFP^; red, CD3). (C) Heatmap of selected cytokines from data in Figure 1A. (D and E) Rank log fold change in expression of cytokines (D) and chemokines (E) in UTI compared with control sorted bladder macrophages. Red bars indicate absolute log fold change of greater than 2. (G) Raw counts of selected cytokines in control bladder macrophages. (F) Correlation of *Il17a* (left) and *Il23a (right)* expression with *Il1b* in murine bladders challenged with PBS (black) or UTI89—24 (blue), 48 (red) and 72 (gray) hours post infection. (H) Efficiency of A647-labeled UPEC phagocytosis by murine bone marrow-derived macrophages with and without prior stimulation with Il17a for 24 h. Flow cytometry gating strategy for macrophages—Live/CD45^+^/CD64^+^/F4/80^+^. Each circle represents a technical replicate (n = 4–6). The 4°C negative control is denoted in blue. Data are representative of three independent experiments. ∗p < 0.05, ∗∗p < 0.01, ∗∗∗p < 0.001, ∗∗∗∗p < 0.0001 by two-way ANOVA with Šídák’s multiple comparisons test (C, H) and linear regression analysis (F). All bladders used were from female mice unless otherwise stated.

Myeloid cell-ILC3 interactions are of critical importance in the gastrointestinal tract, with macrophages producing cytokines such as IL23, IL1β, and TL1A that stimulate ILC3 cytokine production (Longman et al., 2014). In uninfected mouse bladder, we observed a dense network of MNPs, poised to respond to infectious challenge (Figure 6A), with ILC3s and RORγt+ T cells found in close proximity to bladder macrophages (Figure 6B). Following UPEC challenge, there was an increase in *Il1b*, *Il23*, and *Tnfsf15* transcripts in the bladder, with the potential to stimulate type 17 cytokines (Figure 6C).

To determine if macrophages were a source of these transcripts, we flow sorted bladder macrophages and performed bulk RNA-seq. This confirmed a significant increase in *Il1b* at 24 h following infection, with minimal induction of *Il23a* evident (Figure 6D), as well as higher levels of monocyte and T cell chemoattractants *Ccl4* and *Ccl5* (Figure 6E). In keeping with the conclusion that macrophage IL1β is an important activating stimulus for IL17-producing cells, we observed a strong positive correlation between bladder *Il1b* and *Il17* transcripts (Figure 6F). This correlation was less evident with Il23 (Figure 6F). Furthermore, IL23R-deficient mice showed no increase in bladder bacterial load post UPEC challenge, although there was a variable reduction in *Il17* transcripts compared with WT counterparts (Figure S6 and Table S1).

Of note, bladder macrophages also expressed *Csfr2a* and *IL17ra* transcripts (Figure 6G), suggesting a capacity to respond to Th17 cytokines and the potential for cross talk between bladder macrophages and Th17, γδ T cells, and ILC3s. Cellular responses to IL-17A and IL-17F require the ubiquitously expressed IL-17RA paired with the inducible IL-17RC (Toy et al., 2006); *Il17rc* transcripts were also detectable at a lower level in bladder macrophages. To model the potential effects of IL17 on macrophage uptake of UPEC in the bladder, we quantified the phagocytosis of fluorescently labeled UPEC by bone marrow-derived macrophages *in vitro* and observed that the addition of exogenous IL17 augmented bacterial phagocytosis (Figure 6H), suggesting a synergistic relationship between bladder macrophages and ILC3s, and indeed other IL17-producing lymphocytes, whereby IL1β production by bladder macrophages promotes IL17 production by *ILC3s and Th17/*γδ T cells, which in turn acts on macrophages to improve their defensive capabilities.

### scRNAseq of infected bladders maps cytokine production and myeloid interactome of type 17 immune cells

**Figure 7:**
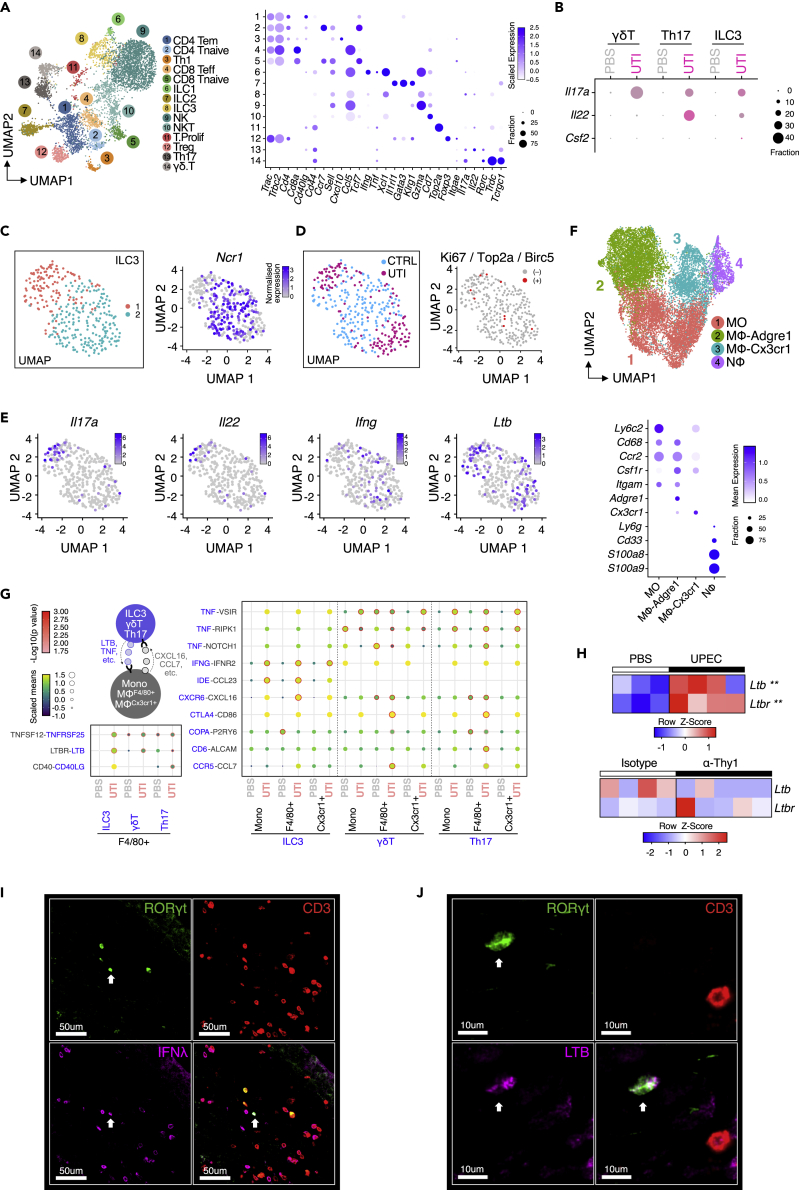
scRNAseq of infected bladders maps myeloid interactome of type 17 immune cells (A) UMAP plot showing integrated analysis of 5,566 T cells isolated from the mouse bladders treated with PBS (n = 10; 3,557 cells) or with UTI89 (n = 10; 2,009 cells). Canonical T cell markers have been identified in each of the populations, showing expression levels in the dot plot. Tem, effector memory T cells; Teff, T effector T cells; Tnaive, naive T cells; Th1, T helper 1; ILC, innate lymphoid cells; NK, natural killer cells; NKT, natural killer T cells; T.Prolif., Proliferating T cells; Treg, regulatory T cells; and Th17, T helper 17 cells. (B) Dot plot showing expression patterns of genes characteristic of ILC3, Th17, and γδT cells. (C) UMAP plot identifying two subpopulations within ILC3s (group 1 in red and group 2 in blue). Cells expressing Ncr1 were indicated by the gradient blue color in the feature plot. (D) UMAP plot showing cells from control (CTRL) or UTI mouse bladders in the ILC3s. Cells expressing proliferation markers Ki67, Top2a, or Birc5 were highlighted in red. (E) Feature plots showing expression levels of the characteristic ILC3 markers *Il17a*, *Il22*, *Ifng*, and *Ltb*. (F) UMAP plot showing integrated analysis of monocytes, macrophages, and neutrophils isolated from the mouse bladders treated with PBS or UTI89. Each population was characterized by a group of canonical markers, shown in the dot plot. (G) Heatmap showing unique ligand-receptor interactions between T cells and myeloid cells in the mouse bladders, treated with or without UTI89. Point size and color correspond to the scaled mean of ligand and receptor genes, analyzed by CellPhoneDB (https://www.cellphonedb.org). Interactions with statistical significance have been highlighted by the red circles. (H) Heatmap of *Ltb* and *Ltbr* transcripts from data in Figures 1A and Figure 5D (upper and lower panels, respectively). Representative confocal image (n = 2) of infected bladder from Rorcγt^GFP^ mouse at 40× demonstrating co-staining of (I) Rorcγt^GFP^ (green); CD3 (red) and Ifnγ (pink) and (J) Rorcγt^GFP^ (green); CD3 (red) and Ltb (pink). (I and J) White arrows in (I) and (J) denote ILC3s. ∗p<0.05, ∗∗p < 0.01, ∗∗∗p<0.001 by two-way ANOVA with Šídák’s multiple comparisons test (H).

To further explore the interaction between type 17 immune cells and MNPs in the bladder beyond Th17 cytokines we performed droplet-based RNA sequencing (scRNAseq) on infected and uninfected bladders using the*10x Genomics* platform. Fourteen clusters of T cells and innate lymphocytes were evident, which we annotated according to canonical marker gene expression, including Th17 cells, γδT cells, and ILC3s (Figures 7A and S7A–S7D). Notably, cells the Th17 cluster showed a marker expression profile associated consistent with a tissue-resident memory T cell phenotype, being *Cd44*+, *Ccr7-,* and *Sell-*negative, with some *Itage* (CD103)+ cells (Figure 7A). Following infection, Th17 cells showed the greatest expression of *Il22* transcripts, with some contribution from ILC3s but little detectable *Il22* in γδT cells (Figure 7B). Induction of *Il17a* post infection was evident in ILC3s, Th17, and γδT cells, with the highest level of expression in ILC3s (Figure 7B). Consistent with this, reactome pathway analysis demonstrated enrichment of “*interleukin signaling”* pathway genes in all three cell types, but particularly prominent in ILC3s, with *Il17a* and *Il17f* among the most enriched in this pathway (Figure S7E). When considering the ILC3 cluster in isolation, two subsets of cells were evident, one that was *Ncr1*-negative and the other containing many *Ncr1*+ cells (Figures 7C and S7D). Both were expanded post UTI and contained *Ki67*/*Top2a*/*Birc5*-positive proliferating cells (Figure 7D). Notably, *Il17* and *Il22* transcripts were largely detectable in the Ncr-negative subset, with *Ifng* in *Ncr1*+ cells (Figures 7E and S7D).

In our dataset, we were also able to identify neutrophils, monocytes, and two broad macrophage clusters, which others have described using flow cytometric analysis (Lacerda Mariano et al., 2020), annotated as F4/80+ or CX3CR1+ (Figure 7F). To investigate how these myeloid cell subsets might interact with type 17 lymphocytes in the bladder we used CellPhone DB, a platform that predicts cellular interactions based on receptor and ligand expression (Vento-Tormo et al., 2018). This identified several additional mechanisms by which these cell types may interact during bladder infection beyond IL17. Notably, *IFNG*-*IFNGR2-*mediated interactions between ILC3s and monocytes and macrophages were significantly increased in the context of UTI, and specific to ILC3s (Figure 7G), consistent with the decrease in *Interferon gamma response* pathway genes we observed in ILC-depleted Rag2 mice (Figure 5I). *Ltb* (encoding lymphotoxin [LT]-β)- and *Tnfsf12* (encoding TWEAK)-mediated interactions were also increased in ILC3s during UTI (Figure 7G). Our analysis of bulk RNA-seq data confirmed a significant increase in *Ltb* in UPEC-infected bladders, with a decrease observed in ILC-depleted *Rag2*^−/−^ mice (Figure 7H). Confocal imaging confirmed the expression of both Ifnγ and Ltb on RORγt-positive, CD3^neg^ ILC3s in mouse bladders (Figures 7I and 7J). In γδ and Th17 T cells, TNF-mediated interactions were increased in UTI, with F4/80+ macrophage secretion of *CCL7* predicted to attract CCR5-expressing γδ and Th17 T cells (Figure 7G). F4/80+ cells also showed increased *CXCL16*-mediated interactions with CXCR6-expressing ILC3, γδ, and Th17 T cells (Figure 7G), identifying tissue macrophages as key orchestrators of type 17 immunity in the bladder.

## Discussion

Previous studies have largely approached the question of which pathways, cytokines, or immune cells are important in bladder infection in a hypothesis-driven manner, measuring or knocking out a favored cytokine (like IL17), or a particular cell type based on prior knowledge cytokine (Jones-Carson et al., 1999; Sivick et al., 2010; Zychlinsky Scharff et al., 2019). Here, we analyzed bulk bladder transcriptomic data in an unbiased way and asked which pathways were most up-regulated in the context of infection, identifying type 17-associated transcripts (including *Il22*, *Il17*, *Rorc*) as the major axis induced post UPEC challenge. Our analysis places this pathway at the heart of the host response to bacterial challenge in the bladder. Notably, IL22 and IL17 have also been detected in human urine in the context of candida UTI (Ahmadikia et al., 2018), supporting the conclusion that this axis may play a similarly important role in human bladder infection. An increase in bladder *Il17* and *IL22* transcripts was evident in both WT and T cell-deficient mice implicating ILC3s as contributors to type 17 responses, in addition to the previously described contribution of γδ T cells to bladder IL17 (Sivick et al., 2010). There is a single previous description of murine bladder ILC3s, which identified CD4^+^ ILC3s (Zychlinsky Scharff et al., 2019), but here we show that NKp46+ ILC3s are also present and we directly profiled their cytokine production and function in the context of bladder infection. Importantly, we also identify ILC3s in human bladders in homeostasis, emphasizing the translational clinical relevance of our data.

Our functional studies using the *Rag2*^−/−^ ILC depletion model, together with the scRNAseq analysis following UPEC challenge, identified IL17 production as the major contribution of ILC3s in acute bladder infection, with a more limited contribution to IL22 production. This presents a marked contrast to the function of ILC3 in the gastrointestinal tract, where IL17 production is not a dominant feature (Lee et al., 2015) and has not been robustly described. However, in the lung, IL17+ ILC3s have been noted in the early stages of injury models (Muir et al., 2016), as we find here in the bladder. In addition to IL17, our analyses also implicated bladder ILC3s as a source of IFNγ and LTβ during acute bacterial cystitis. The importance of ILC-derived IFNγ in shaping bladder defense was further supported by our bulk RNA-seq analysis of ILC-depleted *Rag2*^−/−^ bladders, which showed a reduction in *Interferon gamma response* pathway genes. IFNγ is a canonical Th1 cytokine and, as such, is classically associated with ILC1s. However, intestinal ILC3s exhibit functional plasticity, with signals such as IL-12 and IL-18 promoting up-regulation of T-bet and production of IFNγ by NCR^+^ ILC3 (Klose et al., 2013; Vonarbourg et al., 2010). We found *Ifng*+ *Ncr1*+ ILC3s in our scRNAseq data, and in bulk bladder transcriptomic data *Il18* transcripts were up-regulated post UPEC challenge, providing a signal that may drive bladder ILC3s toward an IFNγ-producing phenotype.

LT signaling is important for the generation of secondary lymphoid organs, including those in the gastrointestinal tract (Alimzhanov et al., 1997; De Togni et al., 1994; van de Pavert and Mebius, 2010), and may also drive tertiary lymphoid structure formation in inflamed tissue (GeurtsvanKessel et al., 2009). ILC3 production of LT is well recognized in NCR^neg^ subsets that include lymphoid-tissue inducer (LTi)-like cells (Klose and Artis, 2016). Notably, ILC3 production of LT has been described in the intestine during *Citrobacter rodentium* infection, and LTβR signaling in intestinal epithelial cells was required for recruitment of neutrophils to the infection site via production of CXCL1 and CXCL2 (Wang et al., 2010). In addition, LTα1β2 and LTβR signaling has been shown to be essential for IL22 production, which subsequently promotes AMP production in epithelial cells, with *Rorc*-expressing cells a major source of LT (Ota et al., 2011; Tumanov et al., 2011). The role of LT signaling in bladder infection has not been previously investigated, although increased LTβR expression has been described in chronic cystitis and in bladder cancer in humans (Shen et al., 2015). Our data implicate ILC3s as an important source of LT during acute infection, with the potential to augment IL22 production and epithelial immune function.

While IL17 has been shown to be important in bladder defense in mouse models, and higher IL17 levels have been identified in infected bladders of female compared with male mice (Zychlinsky Scharff et al., 2019), much less is known about other contributions from type 17 immune cells beyond IL17 production, for example, via IL22 secretion. We identified Th17 cells as the major source of IL22 in the bladder, even in the acute phase of infection 24–48 h after UPEC challenge. This rapid response has the hallmarks of a recall response in memory T cells, and indeed *Cd44* expression was evident in the Th17 cluster in the scRNAseq dataset. IL22R-deficient mice did not show any difference in bacterial numbers in the bladder (although we only examined very early time points, and this may be evident later in the course of infection), but we observed clear effects on AMP production and epithelial cell proliferation. In particular, Reg3γ, Lcn2, and Ptx3 were significantly increased in UTI bladders, and this effect abrogated in IL22R-deficient animals. Previous studies have identified Reg3γ as the most up-regulated AMP in the murine bladder post UPEC challenge and increased concentration of its human orthologue, hepatocarcinoma-intestine-pancreas/Pancreatitis-associated protein (HIP/PAP) in human urine during UTIs (Spencer et al., 2015). However, Reg3γ-deficient mice showed no impairment in bladder UPEC defense in terms of increased bacterial CFUs (Spencer et al., 2015). In contrast, Lcn2-deficient mice had increased bacterial counts in the bladder post UPEC challenge (Steigedal et al., 2014). Similarly, Ptx3-deficient mice also showed increased susceptibility to bacterial cystitis, with Ptx3 capable of opsonizing bacteria to enhance macrophage phagocytosis (Jaillon et al., 2014). This study suggested that Ptx3 secretion by urothelial cells was mediated via cell intrinsic TLR4-MyD88 signaling (Jaillon et al., 2014). However, our data strongly implicate IL22 in the generation of this important AMP. Notably, these studies showing the effects of Lcn2 and Ptx3 deficiency on bladder CFUs examined time points later (day 5) than those used in our study, which may explain why we did not observe a statistically significant difference in bacterial numbers in our experiments with IL22R-deficient mice.

Several publications implicate macrophages in bladder defense, with macrophage depletion associated with an increased bacterial burden in primary infection (Carey et al., 2016; Schiwon et al., 2014) but a reduction in the subsequent development of adaptive immune responses (Mora-Bau et al., 2015). Macrophages have previously been shown to orchestrate neutrophil infiltration in bladder defense (Schiwon et al., 2014); here we find additional functions in stimulating type 17 immune cells via IL1β production. In addition, F4/80-high macrophages were a key source of chemokines that attract CXCR6-expressing ILC3s (CXCL16), as well as CCR5-expressing γδ and Th17 cells (CCL7). Of note, CXCL16 plays a key role in recruiting CXCR6+ NKp46+ intestinal ILC3 in *C*. *rodentium* infection (Satoh-Takayama et al., 2014), identifying an additional facet of cross-tissue similarity in ILC3 biology between gut and bladder. We also found evidence for reciprocal cross talk between ILC3s and macrophage, with IL17 increasing macrophage phagocytosis and impaired maturation of infiltrating monocytes to a tissue macrophage phenotype also evident following ILC depletion, reminiscent of effects observed in gut macrophages in this context (Castro-Dopico et al., 2020).

In summary, our study emphasizes the importance, and provides an overview, of the role of type 17 immunity in bladder defense against bacterial infection, uncovering differing contributions from ILC3, γδT cells, and Th17 cells that inform our understanding of this important clinical condition.

### Limitations of the study

Although our study showed that IL22-dependent AMP transcripts were reduced in *Il22ra* knockout mice compared with WT, CFUs were comparable at the early time points examined (24 and 48 h). However, given previous studies demonstrating the functional importance of AMPs in bladder defense (Jaillon et al., 2014; Steigedal et al., 2014) (as discussed above), we cannot definitively conclude that IL22 plays no important role in bladder defense against bacterial infection. It would be important to examine the impact of *Il22ra* deficiency on bladder bacterial load at later time points following infection, which we were unable to complete owing to practical limitations relating to access to relevant mouse strains. Therefore, further studies will be required to evaluate the temporal effects of IL22 in UTI defense. CFU measurements were performed on single-cell suspensions rather than on cell-disrupted samples, offering the advantage of yielding paired CFU and flow cytometry data from a single experiment. The same protocol was employed across all experiments within this paper enabling a robust comparison between wild type and genetically modified mice.

## STAR★Methods

### Key resources table

**Table:** 

REAGENT or RESOURCE	SOURCE	IDENTIFIER
**Antibodies**		

DAPI (in mounting medium)	Invitrogen	Cat#00-4959-52
LIVE/DEAD	Aqua Invitrogen	Cat#L34957
Hoechst 33,258	Biotium	Cat#40044; RRID: AB_2651133
anti-GFP rabbit polyclonal	Chromotek	Cat#PABG1; RRID:AB_2749857
anti-HLA-DR (AF647)	Abcam	Cat#ab20181; clone TAL 1B5; RRID: AB_445401
anti-mouse F4/80 (AF647)	Abcam	Cat#ab204467; RRID: AB_2810932
anti-mouse F4/80 (FITC)	eBioscience	Cat#11-4801-85; Clone BM8; RRID:AB_2637192
anti-mouse F4/80 (BV605)	Biolegend	Cat#123133; Clone BM8; RRID:AB_465051
anti-mouse Ki67 (PE)	Invitrogen	Cat#12-5698-82; Clone SolA15; RRID:AB_11150954
anti-mouse CD3 (AF488)	Biolegend	Cat#100210; Clone 17A2; RRID:AB_389301
anti-mouse CD3 (PE)	Invitrogen	Cat#12-0031-82; Clone 145-2C11; RRID:AB_465496
anti-mouse CD3 (BV785)	Biolegend	Cat#100231; Clone 17A2; RRID:AB_11218805
anti-mouse CD45 (BV650)	Biolegend	Cat#103151; Clone 30-F11; RRID:AB_2565884
anti-mouse CD45 (FITC)	eBioscience	Cat#11-0451; Clone 30-F11; RRID:AB_465051
anti-mouse CD45 (APC-efluor780)	Invitrogen	Cat#47-0451-82; Clone 30F11; RRID:AB_1548781
anti-mouse CD11b (PerCP-Cy5)	Invitrogen	Cat#45-0112-82; Clone M1/70; RRID:AB_953558
anti-mouse CD19 (PerCP-Cy5.5)	Invitrogen	Cat#45-0199-42; Clone 6D5; RRID:AB_2043821
anti-mouse B220 (PerCP-Cy5.5)	Invitrogen	Cat#45-0452-82; Clone RA3-6B2; RRID:AB_1107006
anti-mouse FCER1 (PerCP-Cy5.5)	Biolegend	Cat#134320; Clone MAR; RRID:AB_10641135
anti-mouse CD4 (PE-Cyanine7)	Biolegend	Cat#100528; Clone GK1.5; RRID:AB_312729
anti-mouse γδTCR (PE-Cyanine7)	Biolgend	Cat#118124; Clone GL3; RRID:AB_11204423
anti-mouse GR1 (ef450)	Invitrogen	Cat#48-5931-82; Clone RB6-8C5; RRID:AB_1548788
anti-mouse Gr1 (APC-eFluor780)	eBioscience	Cat#47-593; Clone RB6-8C5; RRID:AB_1518804
anti-mouse IL22 (APC)	Invitrogen	Cat#17-7222-82; Clone IL22JOP; RRID:AB_10597583
anti-mouse IL17a (BV605)	Biolegend	Cat#506927; Clone TC11-18H10.1; RRID:AB_11126144
anti-mouse Rorγt (BV650)	BD Horizon	Cat#564722; Clone Q31-378; RRID:AB_2738915
anti-mouse 1/A:1/E (AlexaFlor-700)	eBioscience	Cat#56-5321; Clone M5/114.15.2; RRID:AB_494009
anti-mouse CD11c (PE-Cy7)	eBioscience	Cat#25-0114; Clone N418; RRID:AB_2621626
anti-mouse CD11c (PerCP-Cy5.5)	Biolegend	Cat#48-0114; Clone N415; RRID:AB_1548665
anti-mouse Ly6c (APC)	eBioscience	Cat#17-5932; Clone HK1.4; RRID:AB_1724153
anti-mouse CD90.2 (PE-Cyanine7)	Biolegend	Cat#105325; Clone 30-H12; RRID:AB_2303142
anti-mouse CCR6 (BV605)	Biolegend	Cat#129819; Clone 29-2L17; RRID:AB_2562513
anti-mouse NKp46 (PE)	eBioscience	Cat#12-3351; Clone 29A1.4; RRID:AB_1210743
anti-mouse CD64 (BV421)	Biolegend	Cat#139309; Clone X54-5/7.1; RRID:AB_2562694
anti-mouse CD64 (PE)	Biolegend	Cat#139304; Clone x54-5/7.1; RRID:AB_10612740
anti-mouse GATA3 (eFluor660)	eBioscience	Cat#50-9966; Clone TWAJ; RRID:AB_10596663
anti-mouse FcεRIα (PerCP-Cy5.5)	Biolegend	Cat#134320; Clone MAR1; RRID:AB_10641135
anti-human CD3 (AF488)	Biolegend	Cat#300415; clone UCHT1; RRID: AB_389310
anti-human CD3 (PerCP-Cy5.5)	eBioscience	Cat#45-0036; Clone SK7; RRID:AB_1518742
anti-human CCR6 (BV785)	Biolegend	Cat#353422; Clone G034E3; RRID:AB_2563660
anti-human CD11b (FITC)	eBioscience	Cat#11-0118; Clone ICRF4; RRID:AB_1582243
anti-human RORC2 (PE)	R&D	Cat#IC6006P; Clone 600810; RRID:AB_2044720
anti-human BDCA2 (FITC)	Miltenyi Biotec	Cat#130-097-927; Clone 145-2C11; RRID:AB_2661166
anti-human CD11c (FITC)	Biolegend	Cat#301604; Clone 3.9; RRID:AB_314174
anti-human CD123 (FITC)	eBioscience	Cat#11-1239; Clone 6H6; RRID:AB_10854578
anti-human CD34 (FITC)	Biolegend	Cat#343604; Clone 561; RRID:AB_1732005
anti-human CD1a (FITC)	eBioscience	Cat#11-0019; Clone HI149; RRID:AB_1724015
anti-human FcεR1A (FITC)	eBioscience	Cat#11-5899; Clone AER-37; RRID:AB_10732343
anti-human γδTCR (FITC)	eBioscience	Cat#11-9959; Clone B1.1; RRID:AB_465460
anti-human αβTCR (FITC)	eBioscience	Cat#11-9986; Clone IP26; RRID:AB_10854887
anti-human CD94 (APC)	Biolegend	Cat#305508; Clone DX22; RRID:AB_2133129
anti-human CD19 (Alexa-700)	eBioscience	Cat#56-0199; Clone HIB19; RRID:AB_529497
anti-human CD127 (APC-eFluor780)	eBioscience	Cat#47-1278; Clone eBioRDR5; RRID:AB_1548683
anti-human CD14 (eFluor450)	eBioscience	Cat#48-0149; Clone 61D3; RRID:AB_1272120
anti-human CD15 (eFluor450)	eBioscience	Cat#48-0148; Clone H198; RRID:AB_2016661
anti-human c-KIT (BV605)	Biolegend	Cat#313218; Clone 104D2; RRID:AB_2562025
anti-human CD45 (BV650)	Biolegend	Cat#304044; Clone H130; RRID:AB_2563812
anti-human CRTH2 (PE-Dazzle)	Biolegend	Cat#350125; Clone BM16; RRID:AB_2572052
anti-human NKp44 (PE)	Biolegend	Cat#325108; Clone P448; RRID:AB_756100
anti-human CD161 (PECy7)	eBioscience	Cat#25-1619; Clone HP-3G10; RRID:AB_10805517

**Bacterial and Virus Strains**		

UTI89	Professor Scott Hultgren, Department of Molecular Microbiology, Washington University School of Medicine, St. Louis	

**Biological Samples**		

Human bladders	Cambridge Biorepository for Translational Medicine, Cambridge University Hospitals NHS Foundation Trust	N/A

**Chemicals, Peptides, and Recombinant Proteins**		

Percoll GE	Sigma-Aldrich	Cat#17-0891-01
Protease Inhibitor Cocktail	Roche	Cat#4693159001
Buffer RLT Plus	Qiagen	Cat#1053393
AntigenFix	Diapath	Cat#P0016
Penicillin-Streptomycin	Gibco	Cat#15140122
BD Golgi Plug	BD Biosciences	Cat#555029; RRID:AB_2869014
Recombinant Mouse IL-7	BioLegend	Cat#577806
Recombinant Mouse IL-1β	Peprotech	Cat#211-11B
Recombinant Mouse IL-23	Invitrogen	Cat#14-8231-63
*InVivo*MAb anti-mouse Thy1.2 (CD90.2)	BioXcell	Cat#BE0066; Clone 30H12; RRID:AB_1107682
InVivoMAb rat IgG2b isotype control	BioXcell	Cat#BE0090; Clone LTF-2; RRID:AB_1107780
Liberase TM	Sigma-Aldrich	Cat#5401119001
DNase I	Roche	Cat#10104159001
Recombinant murine M-CSF	Peptrotech	Cat#315-02-250UG
Recombinant murine IL-17a	R&D Systems	Cat#421-ML-025
Fetal Bovine Serum	Sigma-Aldrich	Cat#G9665-500ML
Normal rat serum	Avivasysbio	Cat#OOMA00001
Normal mouse serum	Invitrogen	Cat#10410
FcR blocking reagent, human	Miltenyi-Biotec	Cat#130-059-90; RRID:AB_2892112
RNALater solution	Invitrogen	Cat#AM7024

**Critical Commercial Assays**		

TaqMan Gene Expression (Areg)	Thermo Fisher Scientific	Mm01354339_m1
TaqMan Gene Expression (Csf2)	Thermo Fisher Scientific	Mm01290062_m1
TaqMan Gene Expression (Hprt)	Thermo Fisher Scientific	Mm03024075_m1
TaqMan Gene Expression (Il17a)	Thermo Fisher Scientific	Mm00439618_m1
TaqMan Gene Expression (Il22)	Thermo Fisher Scientific	Mm01226722_g1
TaqMan Gene Expression (Lcn2)	Thermo Fisher Scientific	Mm01324470_m1
TaqMan Gene Expression (Reg3γ)	Thermo Fisher Scientific	Mm00441127_m1
TaqMan Gene Expression (Il23a)	Thermo Fisher Scientific	Mm00518984_m1
TaqMan Genotyping Master Mix	Thermo Fisher Scientific	Cat#4371355
TaqMan Fast Advanced Master Mix	Thermo Fisher Scientific	Cat#4444557
RNeasy Micro kit	QIAGEN	Cat#74004
Mouse IL-22 ELISA kit	R&D	Cat#DY582
Mouse IL17a ELISA kit	R&D	Cat#DY421
Mouse GM-CSF ELISA kit	R&D	Cat#DY415
Chromium Single Cell 3' Library & Gel Bead Kit v2	10× Genomics	Cat#PN-120237
Chromium i7 Multiplex Kit	10× Genomics	Cat#PN-120262
Chromium Single Cell 3′ Library & Gel Bead Kit v3	10× Genomics	Cat#PN-1000009
PureLink RNA Mini Kit	Thermofisher	Cat#12183025
TURBO DNase	Thermofisher	Cat#AM2238
Bioanalyzer High Sensitivity DNA Analysis	Applied Biosystems	Cat#5067-4627
GeneChip WT Pico Kit	Thermofisher	Cat#902623
SMARTer Stranded Total RNA-Seq Kit v3 - Pico Input Mammalian	Takara	Cat#634485
TruSeq Stranded Total RNA	Illumina	Cat#20020597
123count eBeads	Thermo Fisher Scientific	Cat#01-1234-42
OCT embedding medium	Thermo Fisher Scientific	Cat#LAMB/OCT

**Deposited Data**		

BMDMs treated with GM-CSF for 24 h	GEO	GSE95404
Gene signatures for macrophages stimulated with IL17a	GEO	GSE20087
Gene signatures for macrophages stimulated with GM-CSF	GEO	GSE95404
Bladder from wild-type mice treated with PBS or UPEC for 24 h	GEO	GSE68220
Gene signatures from murine small intestine ILC1, ILC2 and ILC3s	GEO	GSE85152
Bulk RNA sequencing - IL22ra^−/−^Vs WT UTI bladder (mouse)	this paper	GEO: GSE174735
Bulk RNA sequencing - Rag2^−/−^ + anti-thy1 Vs Rag2^−/−^UTI bladder (mouse)	this paper	GEO: GSE174783
Bulk RNA sequencing - Flow-sorted macrophages (CD45^+^F4/80^+^CD64^+^) from control and UTI89 infected bladders (mouse)	this paper	GEO: GSE174734

**Experimental Models: Organisms/Strains**		

Mouse: C57BL/6J	Jackson Laboratories	RRID: IMSR_JAX:000,664
Mouse: Areg^−/−^ knockout	Prof D. Zaiss, University of Edinburgh, UK	RRID: MGI:5705758
Mouse: *Areg*^flox/flox^ [Areg^tm2a(EUCOMM)Hmgu^]	Prof D. Zaiss, University of Edinburgh, UK	RRID: MMRRC_041447-UCD
Mouse: LysM-cre^+/^ [B6N.129P2(B6)-*Lyz2*^*tm1(cre)Ifo*^/J]	Jackson Laboratories	RRID: IMSR_JAX:018956
Mouse: Rag2 knockout [B6(Cg)-Rag2^tm1.1cgn^/J]	Jackson Laboratories	RRID: IMSR_JAX:008449
Mouse: RORγt knockout [B6.129P2-Rorct^m1litt^/J]	Jackson Laboratories	RRID: IMSR_JAX:007571
Mouse: C57BL/6NJ	Jackson Laboratories	RRID: IMSR_JAX:005304
Mouse: Rorc(γt)-Gfp^TG^	Kind gift from Dr G. Eberl, Institut Pasteur, France	RRID: IMSR_JAX:007572
Mouse: Il22ra1 knockout [Il22ra1^tm1a(EUCOMM)Wtsi^]	Kind gift from Prof G. Dougan, Wellcome Sanger Institute, UK	RRID: MGI:5781643

**Software and Algorithms**		

Cell Ranger (version 2.1.0)	10xGenomics	https://support.10xgenomics.com/single-cell-gene-expression/software/pipelines/latest/using/count
ImageJ	https://fiji.sc	RRID: SCR_003070
GraphPad Prism software version 9.2.0	GraphPad Software	RRID: SCR_002798
CellPhoneDB	Vento-Tormo et al., 2018	RRID: SCR_017054; https://www.cellphonedb.org/
Seurat (version 4.0)	Stuart et al., 2019	RRID: SCR_016341; https://satijalab.org/seurat/get_started.html
R Project for Statistical Computing		RRID: SCR_001905
FlowJo	BD	RRID: SCR_008520
CASAVA (V1.8.2)	Illumina	RRID: SCR_001802
scrublet (V0.2.1)	https://github.com/swolock/scrublet	RRID: SCR_018098
soupx (V1.2.1)	https://github.com/constantamateur/soupx	RRID: SCR_019193
FastQC	Babraham Bioinformatics	UKRRID: SCR_014583
Trim Galore!	Babraham Bioinformatics	RRID: SCR_011847
HISAT2	http://daehwankimlab.github.io/hisat2/	RRID: SCR_015530
RSubread	https://bioconductor.org/packages/release/bioc/html/Rsubread.html	RRID: SCR_016945
DESeq2	https://bioconductor.org/packages/release/bioc/html/DESeq2.html	RRID: SCR_015687
ggplot2	https://cran.r-project.org/web/packages/ggplot2/index.html	RRID: SCR_014601
pheatmap	https://www.rdocumentation.org/packages/pheatmap/versions/0.2/topics/pheatmap	RRID: SCR_016418
GSEA 4.0.1	http://www.broadinstitute.org/gsea/	RRID: SCR_003199
STRING	http://string.embl.de/	RRID: SCR_005223
ssGSEAProjection (v4) - GenePattern	https://www.genepattern.org/modules/docs/ssGSEAProjection/4	
Imaris	Bitplane	RRID: SCR_007370
Adobe Illustrator	Adobe Inc	RRID:SCR_010279

**Other**		

Mm10-3.0.0 mouse genome assembly	NCBI	RefSeq assembly accession: GCF_000001635.20
Illumina Hiseq 4000	https://www.genewiz.com/en-GB	
CLARIOstar spectrophotometer	(BMG Labtech)	
Viia 7 PCR machine	Life Technologies	
TCS SP8 confocal microscope	Leica	RRID:SCR_018169
Zeiss LSM 800 with Airyscan Microscope	Zeiss	RRID:SCR_015963
GentleMACS C tubes	Miltenyi Biotec	Cat#130-093-334; RRID:SCR_020270
GentleMACS Dissociator	Miltenyi Biotec	Cat#130-093-235; RRID:SCR_020267
Precellys Lysing Kit: Hard tissue grinding MK28-R	Precellys	Cat#KT03961-1-008.2

### Resource availability

#### Lead contact

Further information and requests for resources and reagents should be directed to and will be fulfilled by the lead contact, Menna R. Clatworthy (mrc38@cam.ac.uk).

#### Materials availability

This study did not generate new unique reagents.

### Experimental model and subject details

#### Mouse strains

Mouse lines described are either on a C57BL/6J or C57BL/6NJ background as documented. *Rorc(γt)-Gfp*^*TG*^ (transgenic) mice were a kind gift from Dr G. Eberl, Institut Pasteur, Paris, France and *IL22ra1* knockout mice from Prof G. Dougan, Wellcome Sanger Institute, UK. C57BL/6J, C57BL/6JN and *Rag2*^−/−^ (B6(Cg)-Rag2^tm1.1cgn^/J) and RORγt knockout *Rorc(γt)-Gfp/Gfp* (B6.129P2-Rorct^m1litt^/J) mice were obtained from Jackson Laboratories (Margate, UK) and maintained inhouse for several generations. Control mice were co-housed with transgenic mice 2–4 weeks prior to infection models. Female mice aged 8–12 weeks were used for all urinary tract infection models. Male mice aged 8–12 weeks were used for confocal imaging only – this is denoted in the figure legends. Mice were maintained in specific pathogen-free conditions at a Home Office-approved facility in the UK. All murine research was conducted under the Animals (Scientific Procedures) Act 1986 Amendment Regulations 2012 following ethical review by the University of Cambridge Animal Welfare and Ethical Review Body (AWERB).

#### Human samples

Bladder samples was obtained from deceased transplant organ donors after Research Ethics Committee approval (ref 15/EE/0152, East of England Cambridge South Research Ethics Committee) and informed consent from the donor families. 3 male and 2 female donors were included (51–75 years old). Further demographic details are listed in Figure S2C.

#### Microbial strains

Uropathogenic *Escherichia coli* UTI89 was a kind gift from Scott Hultgren, Washington University, USA and was recovered from a patient with acute cystitis. Prior to bladder inoculation *E*.*coli* UTI89 was grown statically in Luria-Bertnai (LB) broth medium for 18 h at 37 °C to ensure type 1-pilius expression as previously described. Bacterial culture was adjusted to an OD_600 nm_ of 0.4–0.5 for the mouse infection.

### Method details

#### *In vivo* urinary tract infection model

Mice were anaesthetised, and residual urine expelled with gentle external pressure. Under aseptic conditions, a polyethelene sheath was inserted into the bladder and 100μL of UPEC or sterile PBS instilled using a 1 mg disposable insulin syringe. Mice were euthanised 24 or 48 hours post infection (as documented) and bladders digested and homogenised to a single cell suspension. Cell homogenates were plated on LB agar and incubated at 37°C overnight for quantification of colony forming units.

#### Murine tissue homogenisation

Prior to euthanasia mice were injected with 1:50 dilution of anti-CD45-A488 antibody (clone: 30-F11, Biolegend) in 200μL sterile PBS to label circulating leukocytes. Mice were left for three minutes before cervical dislocation. Following terminal procedure blood was obtained via cardiac puncture and transferred into an EDTA tube. Bladders were harvested from experimental mice and sliced into approximately 10 mm^3^ pieces and digested for 30 min at 37 °C with agitation, in a digestion solution containing 25 μg/mL Liberase TM (Roche) and 50 μg/mL DNase (Sigma) in 5 mL RPMI (Gibco) in gentleMACS C tubes (Miltenyi Biotec). Samples were then processed using a gentleMACS Dissociator (Miltenyi Biotec) on program spleen 4. The suspension was passed through a 100 μm cell strainer, washed with PBS and blocked with 50:50 mix of normal mouse and rat serum prior to staining. Cell counts per organ were calculated with the addition of 123count eBeads (Invitrogen). For RNA sequencing bladders were divided into two - half placed into RNAlater for 24 h at 4 °C prior to RNA extraction and half homogenised for CFU quantification.

#### Human tissue homogenisation

Human bladder samples were received in ice-cold PBS. The urothelium was stripped from the lamina propria and muscularis layers and sliced into approximately 30 mm^3^ pieces. Samples were digested for 30 min at 37 °C with agitation, in a solution containing 50 μg/mL Liberase TM and 50 μg/mL DNase in 5 mL RPMI in gentleMACS C tubes. Samples were homogenised using a gentleMACS Dissociator on program ‘spleen 4’ and ‘lung 2’. The resulting suspension was passed through a 100μm cell strainer, washed with PBS and pelleted via centrifugation (800xg for 10 min). Lymphocytes were enriched using Percoll 44% gradient at 800xg for 20 min (without break). Cells were then blocked with human FcR block (Miltenyi Biotech). Cell counts per gram were calculated with the addition of 123count eBeads.

#### Flow cytometry

After blocking cells were incubated with live/dead cell staining (Live/Dead Aqua 405, Invitrogen) for 15 min on ice. Cell surface staining occurred on ice for 30 min. Intracellular staining was performed using the FoxP3 intracellular staining kit (eBioscience) as per the manufacturer’s instructions. All samples were acquired on an LSR 4/5 laser Fortessa (BD) and data analysed using FlowJo v10. *Human antibody*: anti-BDCA2- FITC (145-2C11, Miltenyi Biotec), anti-CCR6- BV785 (G034E3, Biolegend), anti-CD11b- FITC (ICRF4, eBioscience), anti-CD11c- FITC (3.9, Biolegend), anti-CD123- FITC (6H6, eBioscience), anti-CD34^−^ FITC (561, Biolegend), anti-CD1a- FITC (HI149, eBioscience), anti-FcεR1A- FITC (AER-37, eBioscience), anti-γδTCR- FITC (B1.1, eBioscience), anti-αβTCR- FITC (IP26, eBioscience), anti-CD94^−^ APC (DX22, Biolegend), anti-CD3^−^ PerCP-Cy5.5 (SK7, eBioscience), anti-CD19^−^ Alexa 700 (HIB19, eBioscience), anti-CD127- APC-eFluor780 (eBioRDR5, eBioscience), anti-CD14^−^eFluor450 (61D3, eBioscience), anti-CD15^−^eFluor450 (H198, eBioscience), anti-c-KIT- BV605 (104D2, Biolegend), anti-CD45^−^ BV650 (H130, Biolegend), anti-CCR6- BV785 (G034E3, Biolegend), anti-CRTH2- PE-Dazzle (BM16, Biolegend), anti-NKp44- PE (P44-8, Biolegend) and anti-CD161- PECy7 (HP-3G10, eBioscience). *Mouse antibody*: Anti-1/A:1/E- AlexaFlor-700 (M5/114.15.2, eBioscience), anti-CD11b- PerCP-Cy5.5 (M1/70, eBioscience), anti-CD11c- PE-Cy7 (N418, eBioscience), anti-CD19^−^ BV785 (6D5, Biolegend), anti-CD3^−^ BV785 (17A2, Biolegend), anti-CD45^−^ FITC (30-F11, eBioscience), anti-F4/80- BV605 (BM8, Biolegend), anti-CD64^−^ BV421 (×54-5/7.1, Biolegend), anti-Gr1- APC-eFluor780 (RB6-8C5, eBioscience), anti-Ly6c- (HK1.4, eBioscience), live/dead fixable Aqua (Thermofisher), anti-CD11b- PerCP-Cy5.5 (M1/70, eBioscience), anti-CD11c- PerCP-Cy5.5 (N415, Biolegend), anti-CD19^−^ PerCP-Cy5.5 (6D5, Biolegend), anti-B220- PerCP-Cy5.5 (RA3-6B2, Biolegend), anti-γδTCR- PerCP-Cy5.5 (GL3, Biolegend), anti-CD3e- PerCP-Cy5.5 (17-A2, Biolegend), anti-FCER1- PerCP-Cy5.5 (MAR1, Biolegend), anti-CD90.2- PE-Cyanine7 (30-H12, Biolegend), anti-CCR6- BV605 (29-2L17, anti-CD90.2- PE-Cyanine7 (30-H12), anti-CD127- APC-efluor780 (A7R34,eBioscience), anti-GR1- ef450 (RB6-8C5, eBioscience), anti-Rorγt-BV650 (Q31-378, Biolegend), anti-NKp46- PE (29A1.4, eBioscience), anti-GATA3- eFluor660 (TWAJ, eBioscience) and anti-GFP rabbit polyclonal (PABG1).

#### IL17a and Il22 cytokine staining

Control (n = 5) and UTI89 infected (n = 5) bladders from C57BL/6 mice were pooled and homogenised into a single cell suspension as described above. Samples were transferred to a U-bottom plate and incubated ex-vivo for 3 h at 37 °C in RPMI supplemented with 10% FCS, 1% Penicillin/Streptomycin, 10mM HEPES, BD Golgi Plug (1:1000, BD Biosciences), IL-7 (10 ng/mL; BioLegend), IL-1β (10 ng/mL; Peprotech) and IL-23 (40 ng/mL; Invitrogen). Samples were blocked with 50:50 mix of normal mouse and rat serum prior to surface staining with live/dead fixable Aqua (Invitrogen), anti-CD11b-PerCP-Cy5.5 (M1/70, Invitrogen), anti-CD11c-PerCP-Cy5.5 (N415, Invitrogen), anti-CD19-PerCP-Cy5.5 (6D5, Invitrogen), anti-B220-PerCP-Cy5.5 (RA3-6B2, Invitrogen), anti-FCER1-PerCP-Cy5.5 (MAR1, Invitrogen), anti-CD4-PE-Cyanine7 (GK1.5, Biolegend), anti-γδTCR-PE-Cyanine7 (GL3, Biolgend), anti-CD45-APC-efluor780 (30F11, Invitrogen), anti-CD3e (12-0031-82, Invitrogen) and anti-GR1-ef450 (RB6-8C5, Invitrogen). Intracellular staining was performed as previously described and staining with anti-IL22-APC (IL22JOP, Invitrogen), anti-IL17a-BV605 (TC11-18H10.1) and anti-Rorγt-BV650 (Q31-378, BD Horizon).

#### *In vivo* ILC3 depletion

Rag^−/−^ mice were given either rat IgG2b isotype control or anti-Thy-1.2 at 0.25mg per mouse (1.25 mg/mL in sterile PBS) via intraperitoneal injection one day prior to and on the day of catheterisation.

#### Phagocytosis assay

Murine femora were flushed with cold PBS through a 100-μm (Falcon) cell strainer. Bone marrow cells were pelleted, resuspended in complete RPMI and plated in a 100 × 20mm Petri dish (Falcon). Cell culture medium was supplemented with 100 ng/mL of macrophage colony stimulating factor (M-CSF, Peprotech) on days 0 and 3. Cells were detached using a cell scraper on day 5/6 and transferred to a 24-well plate at 500,000 cells/well. Cells were incubated for 24 h with recombinant IL-17a (100 ng/mL) or PBS prior to stimulation with UPEC-A647 (UTI89) or PBS for 1 h at 37°C. Control wells were incubated at 4°C to adjust for non-specific binding. Following incubation, cells were washed 3 times with ice-cold PBS. Samples were blocked with 50:50 mix of normal mouse and rat serum prior to surface staining with live/dead fixable Aqua (Invitrogen), anti-CD45^−^ BV650 (30-F11, Biolegend) anti-CD64^−^ PE (×54-5/7.1, Biolegend) and anti-F4/80- FITC (11-4801-85, eBioscience). All samples were acquired on a BD LSR 4 laser Fortessa and data analysed using FlowJo software.

#### Enzyme-linked immunosorbent assay

Whole bladders from control (n = 5) and UTI89 infected (n = 5) bladders from C57BL/6 mice were homogenised in 1 mL of PBS using the Precellys homogeniser system. Samples were then centrifuged at 1500xg for 10 min to remove contaminating material and 750 μL of supernatant taken for ELISA. Quantification of murine IL-22 and GM-CSF on bladder lysates was carried out using commercially available R&D systems Duoset ELISA kits, as per the manufacturer’s instructions. Optical densities were measured at 450 nm and 530 nm background using a CLARIOstar spectrophotometer (BMG Labtech).

#### Flow sorting

Murine bladder macrophages were flow-sorted following UPEC infection or PBS as live CD45^+^F4/80^+^CD64^+^ cells. Cell sorting was performed on 5 laser Synergy (Sony Biotechnology Inc.) into 500uL of RLT lysis buffer (Qiagen).

#### Immunofluorescence microscopy

Murine bladders were fixed in AntigenFix for 30 min and human bladder in 1% PFA overnight at 4 degrees. Samples were then rinsed in PBS for 5 min and transferred into 30% sucrose in PBS for 24 h. 30μm sections were permeabilized and blocked-in blocking buffer containing 0.1M TRIS, 0.1% Triton, 1% normal mouse serum, 1% normal rat serum, 1% BSA for 1h at room temperature. Staining was performed in blocking buffer for 2h at room temperature prior to washing in PBS and mounting in Fluoromount-G or Fluoromount-G with DAPI. When required, a secondary staining was performed in blocking buffer for 2h at room temperature prior to washing and mounting. Images were acquired using a TCS SP8 confocal microscope and raw images were processed using Imaris. *Human antibody*: anti-RORC2- PE (IC6006P, R&D); anti-CD3^−^ AF488 (UCHT1, Biolegend); anti-HLA-DR- AF647 (ab223907, Abcam) and Hoechst 33342 (29 hermofisher). *Mouse antibody*: anti-F4/80- AF647 (ab204467, Abcam); anti-GFP rabbit polyclonal (PABG1, Chromotek); anti-CD3^−^ AF488 (17A2, Biolegend); anti-CD3^−^ PE (145-2C11, Invitrogen) and anti-Ki67- PE (SolA15, Invitrogen). *Dyes*: Flash Phalloidin 488 (Bio- Legend), Hoechst 33258 (cat# 40044, Biotum), DAPI (in mounting medium, cat# 00-4959-52, Invitrogen).

#### RNA extraction and reverse transcription

RNA was extracted from either whole tissue or cell suspension. Whole tissue samples were homogenised in 1 mL of RNA lysis buffer using the Precellys homogeniser system. Samples were then centrifuged at 1500xg for 10 minutes to remove contaminating material and 750 μL of supernatant taken for RNA extraction. RNA extraction was performed using the Ambion RNA PureLink kit (Life Technologies) per the manufacturer’s instructions. For expected low RNA yield the Rneasy Plus Micro kit (Qiagen) was used. RNA was quantified using NanoDrop spectrophotometer (ThermoFisher). Complementary DNA (cDNA) was prepared using SuperScript IV VILO Master Mix (Invitrogen) and amplified with BioRad PCR machine (BioRad). RT-PCR was performed using TaqMan 2× Fast Master Mix (Applied Biosystems) on the Viia 7 PCR machine (Applied Biosystems).

#### Quantitative polymerase chain reaction

All qPCR was carried out in triplicate with Taqman reagents and the following pre-designed TaqMan Gene Expression Assay primers and probes (Thermo Fisher Scientific). Murine primers: *Areg* (Mm01354339_m1), *Csf2* (Mm01290062_m1), *Hprt* (Mm03024075_m1), *Il17a* (Mm00439618_m1), *Il22* (Mm01226722_g1), *Il23a* (Mm00518984_m1), *Lcn2* (Mm01324470_m1), *Reg3γ* (Mm00441127_m1). qPCR was performed on the Viia 7 PCR machine (Life Technologies). Gene expression was normalized to Hprt using the 2^−ΔCt^. The 2^−ΔΔCt^ method was used for normalization between experimental conditions and genotypes.

#### RNA sequencing sample preparation

##### Bladder macrophage RNAseq

Flow-sorted macrophages (CD45^+^F4/80^+^CD64^+^) from control and UTI89 infected bladders were immediately lysed in 500μL RLT plus buffer (QIAGEN). Samples were vortexed, snap frozen on dry ice and stored at −80 °C. RNA was extracted from cell lysates using the Rneasy plus micro kit (QIAGEN) as per the manufacturer’s instructions. Optimal DNA depletion columns (QIAGEN) were used to remove contaminating genomic DNA. Purified RNA was eluted in nuclease free water (Ambion) and stored at −80 °C. Quality and concentration of the purified RNA was assessed using an RNA pico chip (Applied Biosystems) using a Bioanalyzer 2000 (Applied Biosystems) as per the manufacturer’s instructions. For all RNAseq experiments, samples had an RNA integrity number >8. For the preparation of libraries, SMARTer stranded total RNAseq mammalian pico input kit (Takara) was used as per the manufacturer’s instructions. To produce the libraries, 5ng of total RNA was used. Sequencing was carried out on Hiseq 4000 on a 2 × 150bp sequencing run. These data have been deposited under GSE174734.

##### IL22ra^−/−^ vs WT UTI bladder RNAseq

Four *IL22ra*^*−/−*^ and C56BL/6J bladders 24 h after challenge with UTI89 were bisected and placed directly into RNAlater for 24 h at 4 °C. Samples were homogenised and RNA extracted as described in “RNA extraction and reverse transcription” and contaminating DNA digested with TurboDNase (Invitrogen). Quality of RNA was checked as above. RNAseq libraries were prepared using Illumina TruSeq Stranded total RNA library prep kit with 1ug of RNA as per manufacturer’s instructions. Library size was assessed with a High Sensitivity DNA chip (Applied Biosystems) using a Bioanalyzer 2000 (Applied Biosystems) as per the manufacturer’s instructions. Sequencing was carried out on Hiseq 4000 on a 2 × 150bp sequencing run. These data have been deposited under GSE174735.

##### Rag2^−/−^ + anti-thy1 Vs Rag2^−/−^ UTI bladder RNAseq

Rag2^−/−^ + anti-Thy1 (n = 4) and Rag2^−/−^ + isotype (n = 4) bladders 24 h after challenge with UTI89 were bisected and placed directly into RNAlater for 24 h at 4 °C. Samples were processed as above. These data have been deposited under GSE174783.

#### RNA sequencing and analysis

All sequencing was carried out at Genewiz (NJ, USA). Pooled libraries were de-multiplexed by Genewiz using Casava (Illumina) before transfer of the data to the University of Cambridge. The Fastq files from libraries prepared using the Takara library prep were trimmed of the first 3 nucleotides of the R1 strand and contaminating adaptor sequences and poor-quality bases removed (bases with a phred 33 score of <30) using trimgalore! (Babraham bioinformatics). The Illumina library preps were only trimmed for quality. Sequencing quality of the resulting files was assessed using FastQC (Babraham bioinformatics). Fastq files were aligned to the mm10 genome using hisat2. Subsequent analysis was carried out in R. Reads were counted and assigned to genes using the Featurecount function from the RSubread package. Differential expression analysis was carried out using DESeq2 using a linear model with an appropriate design matrix following the default workflow. Resulting figures were plotted using ggplot2 and pheatmap. GSEA was performed for RNA-seq data by first assigning a rank metric to each gene. GSEA was then run using GSEA 4.0.1 using the pre-ranked option with the classic setting against either gene sets from the molecular signature database or custom 100/200-gene gene sets indicated in the text. STRING analysis was performed using the online portal (https://string-db.org) selecting high-highest confidence parameters (0.700–0.900) with no additional interactors, unless stated in the text. Gene signatures for macrophages stimulated with GM-CSF or IL17a were created using differential expressed genes (p < 0.05 and LFC >2) from publicly available datasets; GEO: GSE95404 (Ao et al., 2017) and GEO: GSE20087 (Zhang et al., 2010) respectively. Single sample GSEA (ssGSEA) v4 was used to calculate enrichment scores for curated GM-CSF and IL17a signatures.

#### Public microarray

Publicly available microarray datasets were downloaded from GEO (https://www.ncbi.nlm.nih.gov/geo/) along with appropriate chip annotation data. All analyses were carried out using R. Data was normalized using RMA and limma. Probes were reduced to one probe per gene by selecting the probe with the greatest variance across the samples using the gene filter package. Differential expression was varied out using limma with an appropriate design matrix. Public datasets used in this study are as follows: GEO:GSE68220 (Bladders from wild-type mice treated with PBS or UPEC for 24 h); GEO:GSE95404 (BMDMs treated with GM-CSF for 24 h) and GEO:GSE20087 (BMDMs treated with IL17a for 12 and 24 h).

#### Single cell RNA sequencing

N = 5 bladders from C57BL/6J mice catheterised with UTI89 for 24 h and N = 5 bladders from naive mice were pooled before processing to a single cell suspension as described earlier (see ‘Murine tissue homogenisation and intravascular labelling of circulating leukocytes’). Cell suspensions were counted using a hemocytometer and adjusted to 1 × 10^6^ cells/mL. Two lanes per condition were loaded according to the standard protocol of Chromium single 3’ (V2 chemistry) to capture 20,000 cells/channel. Libraries were prepared according to the manufacturer’s protocol, followed by Bioanalyzer quality checks. Sequencing was performed on an Illumina Hiseq 4000.

Data was processed using the Cell Ranger 3.0.0 pipeline (10× Genomics). The FASTQ files were then aligned to the mouse genome reference sequence, mm10. The processed data was analysed using Seurat 3.2.0, doublets (Stuart et al., 2019) were detected with DoubletFinder 2.0.2 and removed, and multi-sample integration was performed with canonical correlation analysis. Cells with >250 and <2500 genes, > 1000 UMIs, and <10% mitochondrial genes were maintained. UMI counts, mitochondrial and ribosomal genes, and cell cycle phase scores were subtracted during data scaling. T cells or monocytes and macrophages compartments were isolated and re-integrated for further investigation in this study. GSEA was performed in the clusterProfiler package and was visualised with the GOChord function in the GOplot package. Briefly, genes were ranked according to their log2 expression levels (UTI vs PBS) in a descending order and were analysed with GSEA for Reactome database. The leading-edge genes from the significantly enriched immune pathways (p < 0.05, NES >0) were then subject to visualisation. Ligand-receptor analysis was performed with CellPhoneDB (Vento-Tormo et al., 2018) and was visualised with the plot_cpdb function in the ktplots package. The normalised counts and meta data extracted from Seurat objects were applied for the statistical analysis from CellPhoneDB in python 3.7.9. The resulting p values and means were then filtered and visualised with ktplots.

### Quantification and statistical analysis

Statistical analysis was performed using GraphPad Prism software, R, IPA, or GSEA and have been described in the relevant methods sections and figure legends accordingly. For *in vitro* stimulation experiments, mean ± standard error of mean (SEM) is shown. For RNAseq bioinformatics analyses, p values were calculated using the standard DE-Seq 2 method with multiple correction using BH procedure. For microarray experiments, p values were calculated using the limma package with multiple correction using BH procedure. ^∗^ p < 0.05; ^∗∗^ p < 0.01; ^∗∗∗^ p < 0.001; ^∗∗∗∗^ p < 0.0001. Sample sizes (n) for all shown data can be found in figure legends. *In vitro* stimulations were performed in triplicate, unless stated, and sample sizes for *in vivo* experiments were determined based on initial experiments.

## Data Availability

• Bulk RNA-seq data have been deposited at GEO and are publicly available as of the date of publication. Accession numbers are listed in the key resources table. This paper also analyzes existing, publicly available data and accession numbers are listed in the key resources table. Single-cell RNA-seq data is available from the lead contact upon request. This paper does not report original code. • Microscopy data reported in this paper will be shared by the lead contact upon request. • Any additional information required to reanalyze the data reported in this paper is available from the lead contact upon request.

## Supplemental information

Document S1. Figures S1–S7

Table S1 [related to figure 3–5] – Raw colony forming units for all experiments
