# Supplementary material for: Group 3 innate lymphocytes make a distinct contribution to type 17 immunity in bladder defence
Source: iScience. 2022 Jun 22;25(7):104660. doi: 10.1016/j.isci.2022.104660 (PMC9283510; doi:10.1016/j.isci.2022.104660)
Supplement: Document S1. Figures S1–S7 [file mmc1.pdf]

iScience, Volume *25*

Supplemental information

Group 3 innate lymphocytes make a distinct contribution to type 17 immunity in bladder defence

Alexandra M. Riding, Kevin W. Loudon, Andrew Guo, John R. Ferdinand, Laurence S.C. Lok, Nathan Richoz, Andrew Stewart, Tomas Castro-Dopico, Zewen Kelvin Tuong, Remi Fiancette, Georgina S. Bowyer, Aaron Fleming, Eleanor S. Gillman, Ondrej Suchanek, Krishnaa T. Mahbubani, Kourosh Saeb-Parsy, David Withers, Gordan Dougan, Simon Clare, and Menna R. Clatworthy

Figure S1 - related to Figure 1

S1A

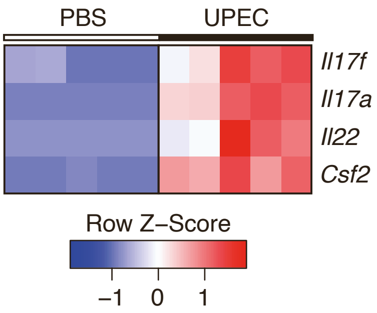

S1B

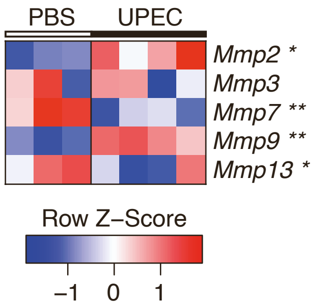

S1C

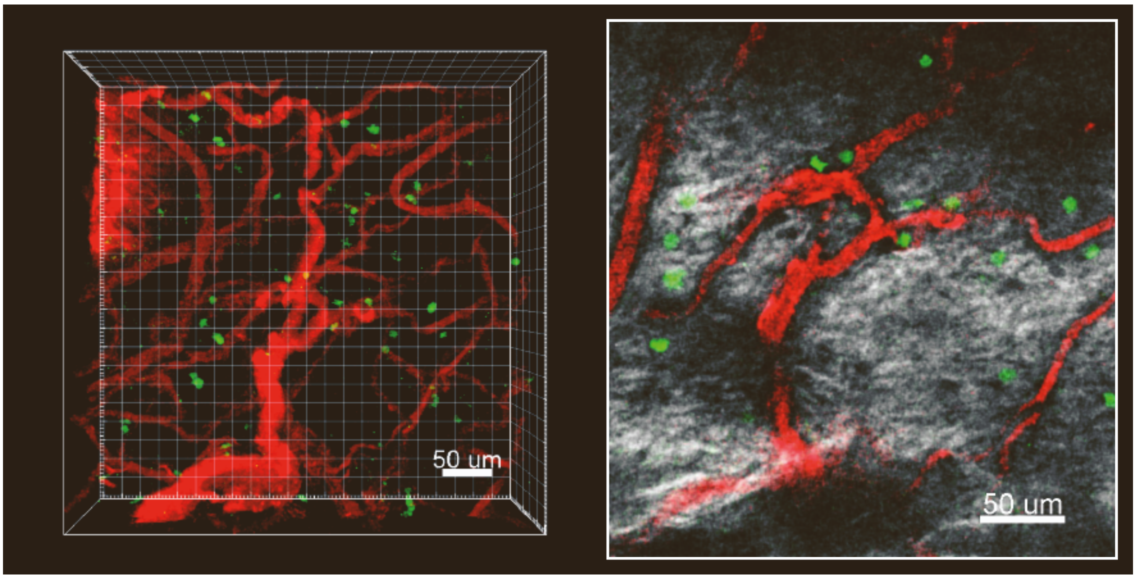

S1D

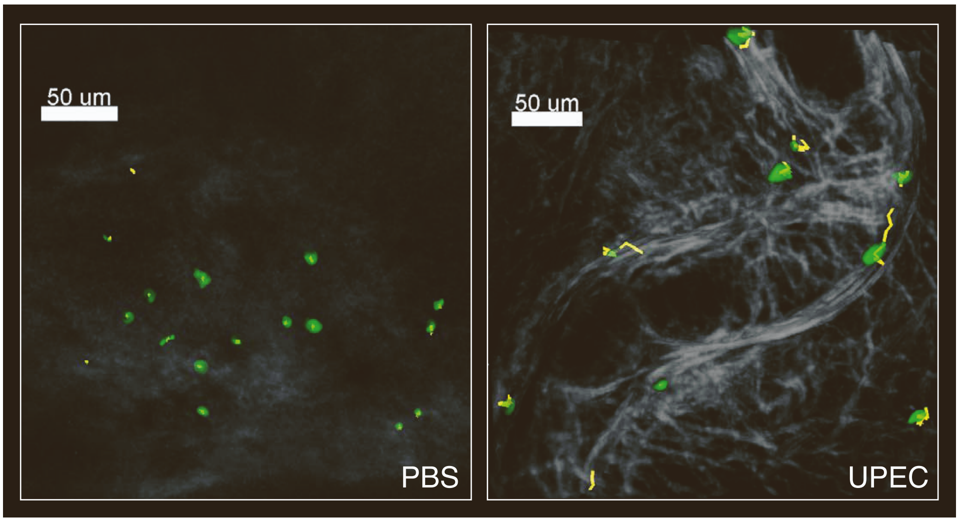

Figure S1 - Th17 cytokine and MMP expression in UTI by RNA sequencing and still frames from intravital imaging of Rorγt-GFP bladder in PBS or UPEC treated bladders, related to Figure 1. (A) Heat map of Th17 cytokines in bladders catheterised with PBS or UPEC from GSE68220. (B) Heatmap of selected IL17 induced MMPs from data in figure 1A. *P < 0.05, **P < 0.01, ***P < 0.001, ****P < 0.0001 by two-way ANOVA with Šídák's multiple comparisons test. (C) Still frame from intravital image of Rorγt-GFP bladder following intravenous administration of Qtracker (labelling blood vessels) showing location of GFP positive cells. Second harmonics (white) and Rorγt-GFP cells (green). (D) Still frame from intravital image of Rorγt-GFP bladder 24 hours following catheterisation with PBS or UPEC with cell tracks shown (Rorγt-GFP cells - yellow). Second harmonics (white) and Rorγt-GFP cells (green).

Figure S2 - related to Figure 2

S2A

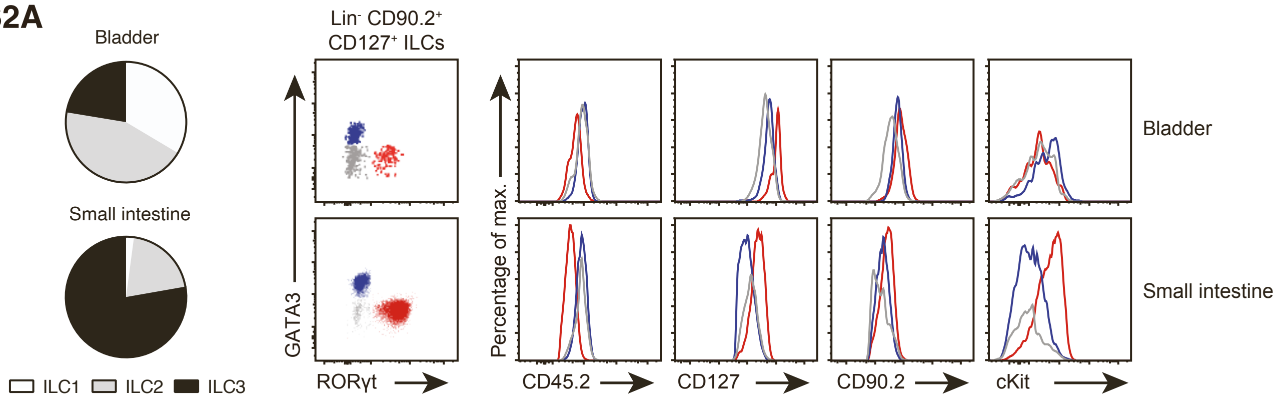

S2B

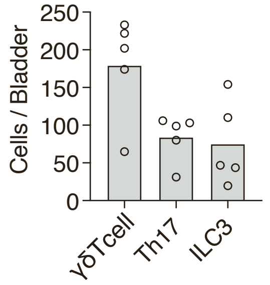

S2C

Organ	Age	Sex	DCD/DBD	Cause of Death
Bladder	75	F	DCD	ICH
	68	F	DCD	ICH
	55	M	DBD	ICH
	51	M	DCD	Trauma RTC/ ICH
	63	M	DCD	ICH

S2D

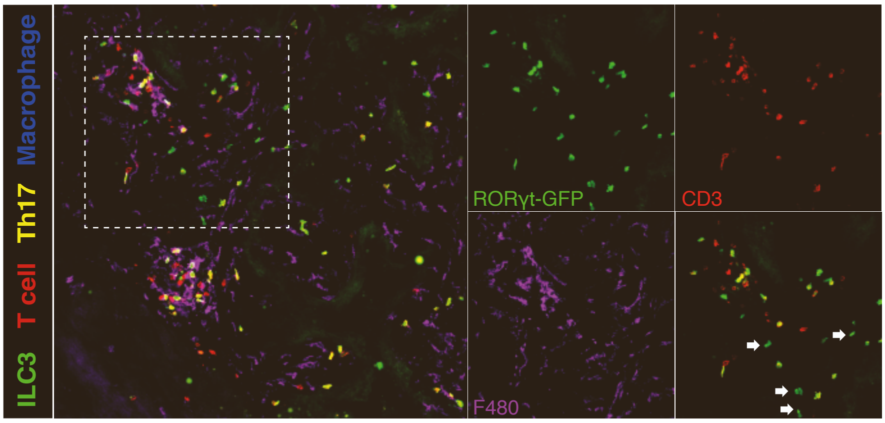

S2E

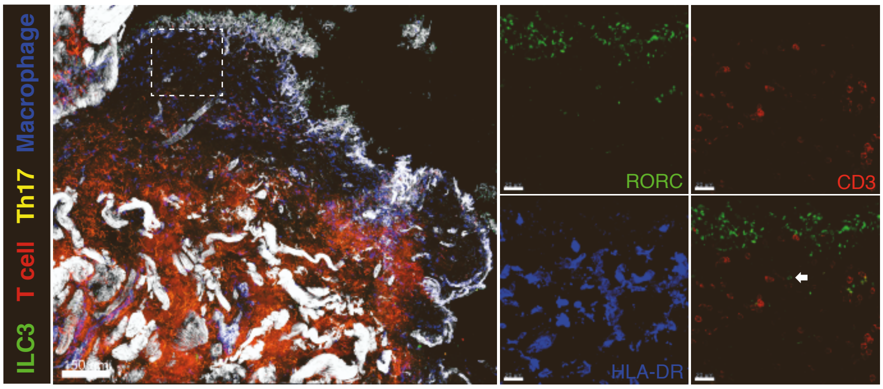

S2F

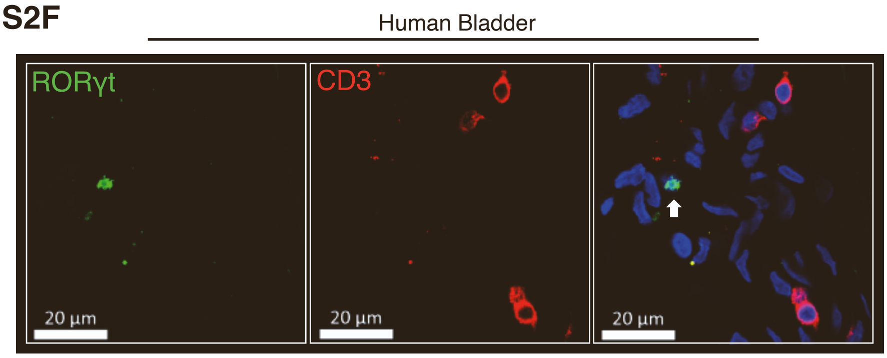

S2G

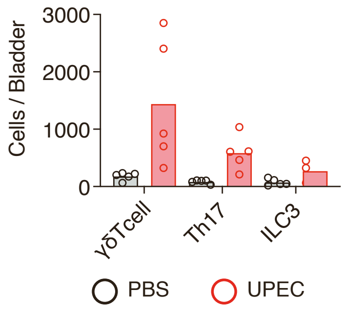

Figure S2 - ILCs, γδT cells and Th17 cells in naïve and infected bladders, sample demographics of human tissue and confocal microscopy of murine and human bladders, related to Figure 2. (A) Pie chart of ILC proportions (left) and histograms of selected surface markers in naive bladders and small intestine. ILC1 - grey, ILC2 - red and ILC3 - red. Gating strategy - Live/CD45+/Lineage-/CD90.2+/CD127+. (B) ILC3, Th17 and γδT cell numbers in uninfected bladders. Each dot represents an indiviual bladder. (C) Demographic details of deceased donor bladders (figure 2C). DCD - donation after circulatory death; DBD - donation after brainstem death; ICH - intracerebral haemorrhage and RTC - road traffic collision. (D) Representative confocal image (expanded from figure 2D main text) of naive bladder from RorcγtGFP mouse at 40X (green, RorcγtGFP; red, CD3; magenta, F480). (E) Confocal image of human female bladder (expanded from figure 2D main text) from deceased donor at 40X (green, RORC; red, CD3; blue, DAPI). (F) Confocal image of human male bladder from deceased donor at 40X (green, RORC; red, CD3; blue, DAPI). ILC3s - green, T cells - red, yellow - Th17 and macrophages - magenta. White arrows in denote ILC3s. (G) ILC3, Th17 and γδT cell numbers in control (grey) and UTI (red) bladders. Each dot represents an indiviual bladder.

Figure S3 - related to Figure 3

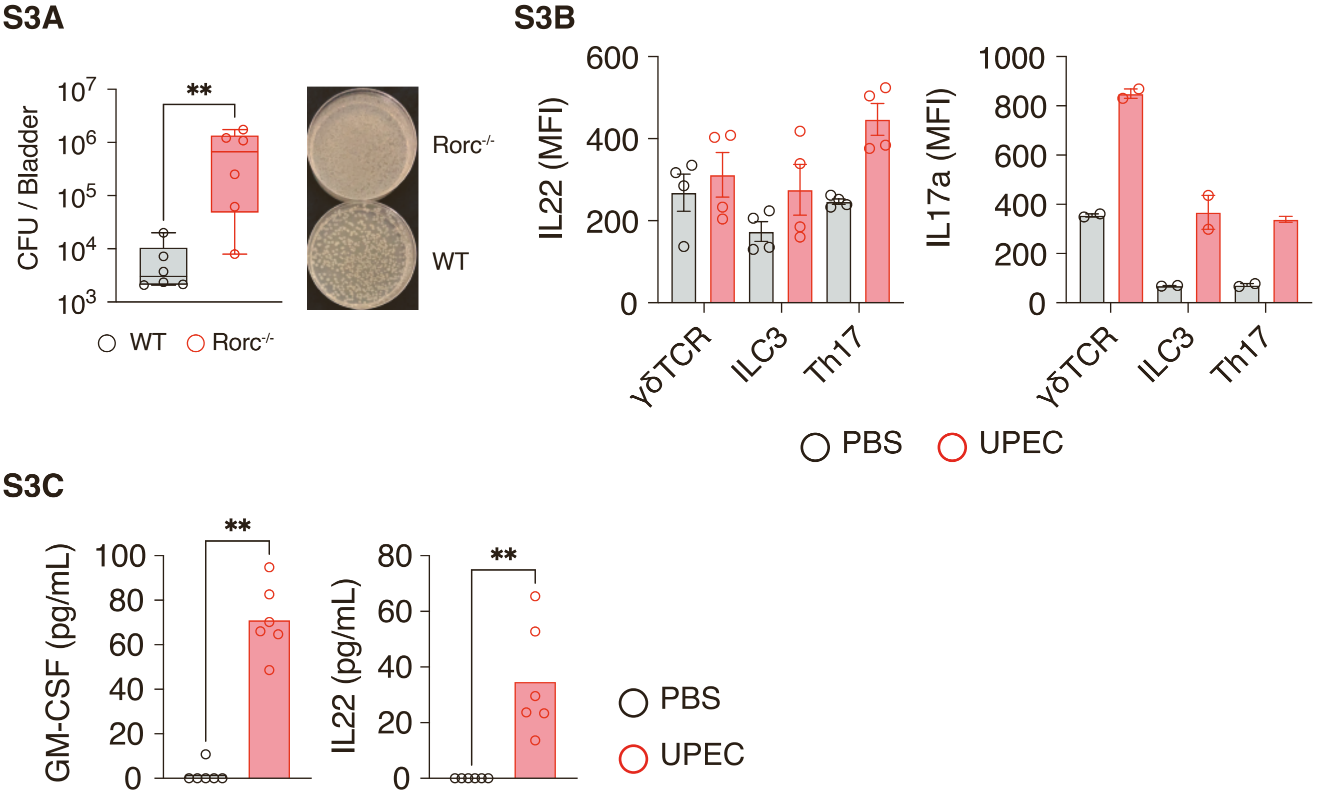

Figure S3 - Validation UTI in *Rorc*-deficient mouse and Th17 cytokine protein expression by ELISA and flow cytometry, related to Figure 3. (A) CFUs per bladder 48 hours following UTI in a replicate experiment (n=6 per group) in C57BL/6 (black circle) and Rorc-/- (red circle) (left panel) and corresponding image of bacterial growth on agar plates; 1:300 dilution (right panel). (B) Quantification of IL22 and IL17a (intracellular staining) MFI in selected immune subsets from murine bladder 24 hours post UTI (red circle) or PBS control (black circle) for data shown in Fig 2D. Each circle is a combination of 3 murine bladders. N=6 per group. (C) GM-CSF and IL22 ELISA on supernatants from whole bladder homogentes from mice treated with PBS or UTI89 (n=6 per group). Mean ± SEM indicated. Each point represents a biological replicate. **P < 0.01 by Mann-Whitney test.

Figure S4 - related to Figure 4

S4A

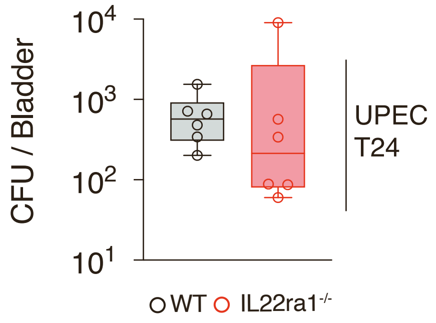

S4B

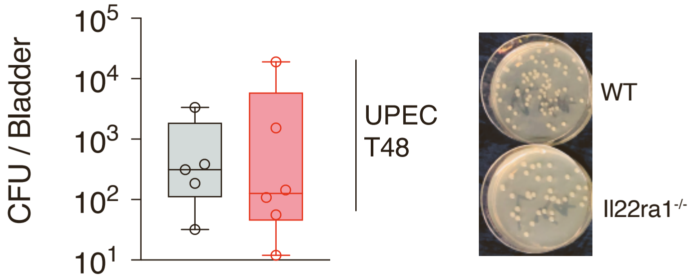

Figure S4 - Validation UTI at 24 and 48 hours IL22ra1$^{-/-}$ mice, related to Figure 4. (A) CFUs per bladder 24 hours following UTI in a replicate experiment (n=6 per group) in C57BL/N (black circle) and IL22ra1-/- (red circle) (left panel) and corresponding image of bacterial growth on agar plates; 1:30 dilution (right panel). (B) CFUs per bladder 48 hours following UTI (n=5-6 per group) in C57BL/N (black circle) and IL22ra1-/- (red circle). Each point represents a biological replicate. *P < 0.05, by Mann-Whitney test.

Figure S5 - related to Figure 5

S5A

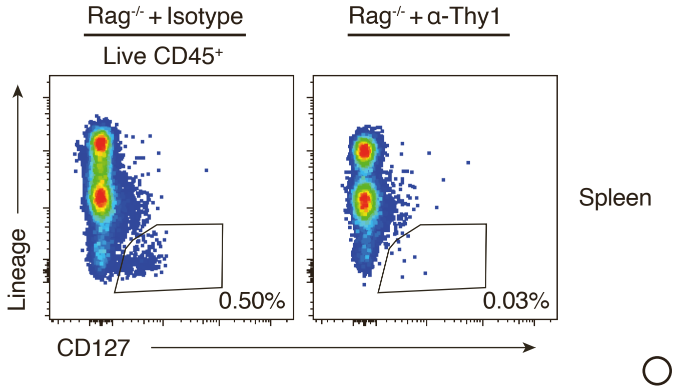

S5B

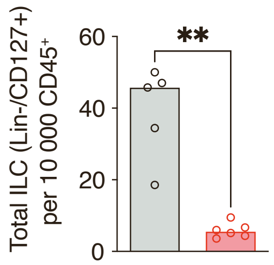

S5C

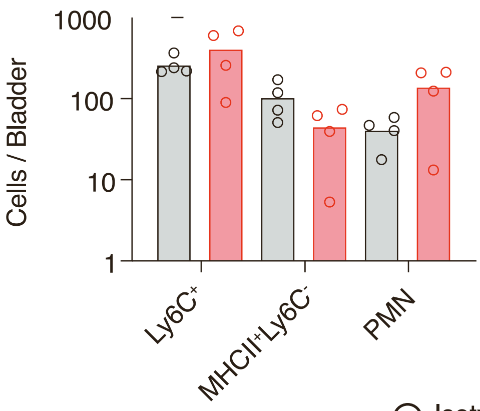

S5D

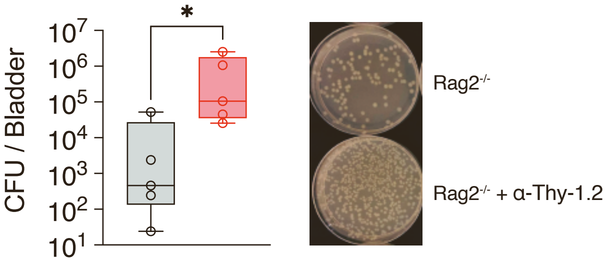

Figure S5 - Validation UTI in *Rag2*$^{-/-}$ with and without ILC-depletion and confirmation of ILC3 depletion using flow cytometry, related to Figure 5. (A) Representative flow cytometry plots and (B) quantification of total splenic ILCs in Rag-/- mice following treatment with isotype control or anti-Thy1 antibody confirming effective depletion. Gating for ILCs - Live/CD45+/lineage-/CD127+. (C) Quantification of selected immune subsets in Rag2-/- mouse bladders following anti-thy1.2 antibody depletion (red circle) or isotype control (black circle). (D) CFUs per bladder 24 hours following UTI in a replicate experiment (n=5 per group) in Rag2-/- mouse bladders following anti-thy1.2 antibody depletion (red circle) or isotype control (black circle) (left panel) and corresponding image of bacterial growth on agar plates; 1:400 dilution (right panel). Each point represents a biological replicate. *P < 0.05, **P < 0.01 by Mann-Whitney test.

Figure S6 - related to Figure 6

S6A

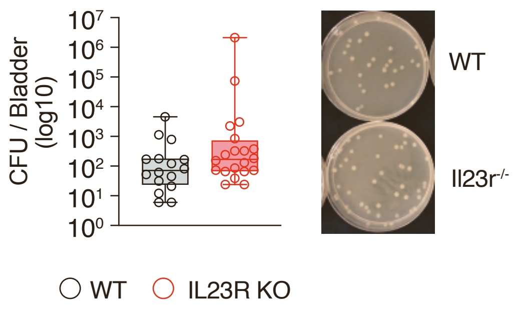

S6B

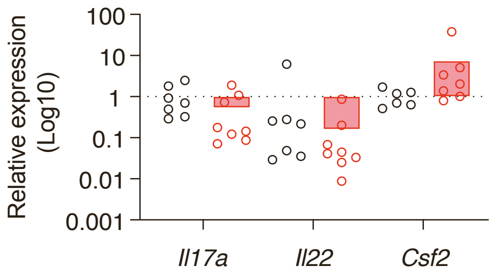

S6C

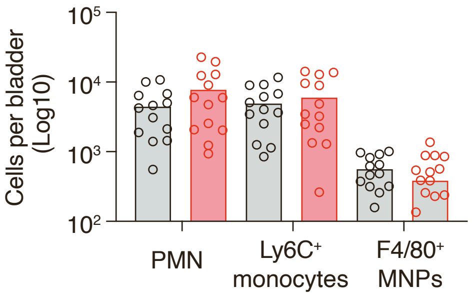

S6D

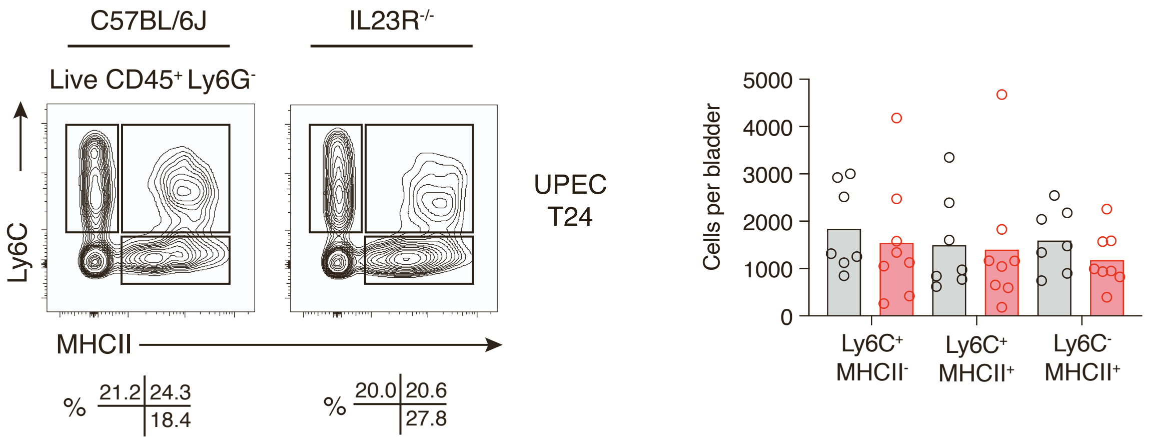

Figure S6 - IL23r$^{-/-}$ mice demonstrate no difference in cystitis severity, Th17 cytokines or immune cell subsets following UTI, related to Figure 6. (A) Colony forming units per bladder 24 hours after infection with UTI89 in C57BL/6J (grey) and IL23r-/- (red) mice (left panel) and corresponding image of bacterial growth on agar plates; 1:3 dilution (right panel). Graph represents combination of 3 experiments (n=4-6 per group). (B) qPCR of Th17 cytokines in C57BL/6J and IL23r-/- bladders 24 hours post infection. Results relative to C57BL/6J bladders. Data are representative of 2 independent experiments. (C) Quantification of absolute cells counts in C57BL/6J and IL23r-/- bladders 24 hours post infection (n=4-6 per group) for the indicated subsets. (D) Bladder “monocyte waterfall” subset quantification by flow cytometry 24 hours post infection with UTI89 in C57BL/6J and IL23r-/- bladders (n = 4-6 per group). Flow plots of CD45+Ly6G-CD11b+CX3CR1+ waterfall subsets (left) and quantification of absolute cell counts for the indicated subsets (right) are shown.

Figure S7 - related to Figure 7

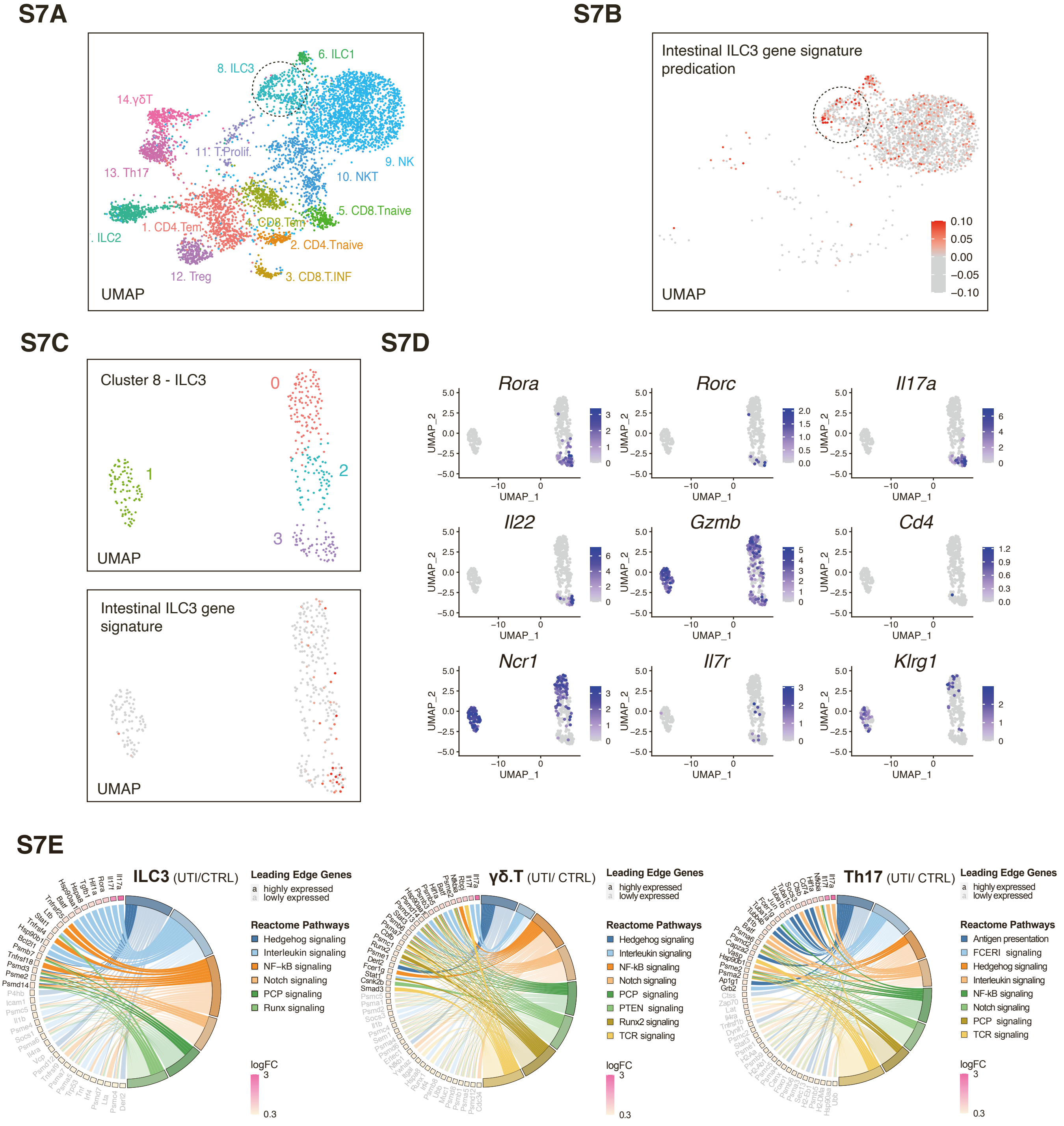

Figure S7 - Computational validation of bladder ILC3 identity and enrichment pathways in UTI, related to Figure 7. (A) UMAP plot showing integrated analysis of 5,566 T cells/innate lymphocytes isolated from mouse bladders treated with PBS (n=10; 3,557 cells) or with UTI89 (n=10; 2,009 cells). ILC3 cluster denoted by an interrupted circle. (B) Intestinal ILC3 (curated from Gury-BenAri et al, Cell 2016; GEO: GSE85152) module summary score. ILC3 cluster denoted by an interrupted circle. (C) UMAP of re-clustered "8.ILC3" from data in (A) and enrichment of intestinal ILC3 module summary score within these clusters. (D) Feature plot of selected ILC3 genes for data in (C). (E) Circos plots showing enriched pathways and their leading-edge gene expression upon UTI. Ribbon colours correspond to each of the enriched pathways. Ribbons with the same colours connect with leading-edge genes identified in certain pathways.
